# Structural and dynamic functional controllability: shared variance between network control metrics limits attribution to distinct domains of intelligence

**DOI:** 10.3389/fnhum.2026.1920417

**Published:** 2026-10-02

**Authors:** Justin Ng, Jamie D. Feusner, Colin Hawco

**Affiliations:** 1 Campbell Family Mental Health Research Institute, Centre for Addiction and Mental Health, Toronto, ON, Canada; 2 Institute of Medical Science, University of Toronto, Toronto, ON, Canada; 3 Department of Psychiatry, University of Toronto, Toronto, ON, Canada; 4 Department of Women’s and Children’s Health, Karolinska Institutet, Stockholm, Sweden

**Keywords:** crystallized intelligence, dMRI, dynamic functional connectivity, fluid intelligence, fMRI, general intelligence, network control theory, structural connectivity

## Abstract

**Introduction:**

Fluid and crystallized intelligence have recently been hypothesized to reflect the brain network’s capacity to facilitate transitions into hard-to-reach, high-energy states, measured via modal controllability, and easy-to-reach, low-energy states, measured via average controllability, respectively. Controllability is typically measured for the structural connectome, but dynamic functional connectivity may better capture temporally varying processes relevant to cognition.

**Methods:**

Using data from the Human Connectome Project, we compared the prediction of crystallized and fluid intelligence from controllability metrics measured in both structural and dynamic functional connectivity. We hypothesized dynamic functional connectivity would outperform structural connectivity, and modal and average controllability would differentially predict fluid and crystallized intelligence.

**Results:**

As expected, dynamic functional connectivity outperformed structural connectivity in predicting cognitive performance. However, modal and average controllability displayed similar predictive power for fluid and crystalized intelligence, and performed comparably to regional (or node) strength, a simpler graph metric reflecting the strength of immediate regional connections that strongly covaries with both controllability measures. Furthermore, controllability most strongly predicted general intelligence, the shared factor between crystallized and fluid intelligence. General intelligence was associated with higher average controllability (and greater node strength) in transmodal regions and higher modal controllability (and lower node strength) in unimodal regions in dynamic functional connectivity.

**Discussion:**

These findings clarify how network control maps onto cognitive ability across the cortical hierarchy, while suggesting that shared variance between controllability metrics hinders specific attribution to crystallized and fluid intelligence, challenging prior hypotheses.

## 1 Introduction

Intelligence refers to the tendency of individuals to have a higher general cognitive capacity across tasks (e.g., learning, reasoning, and understanding), thereby supporting success in life (Gottfredson, 1997). This observation is captured by the positive correlation in performance across cognitive measures (Spearman, 1904). Factor analysis reveals key domains of intelligence, notably general (g), fluid (gF), and crystallized cognition (gC) (Cattell, 1963). While g describes correlations across all tests, gF relates to problem-solving and adaptation in new situations without prior experience, and gC relates to the accumulation and application of knowledge. gF and gC may have different bases. For example, gF peaks in adulthood and declines with age, while gC is more stable (Horn et al., 1981). gF is associated with adaptive cognitive control and maintenance of task goals, supported by frontoparietal (FPN) and cingulo-opercular networks (CON) (Dosenbach et al., 2008), whereas gC is associated with semantic and episodic memory, supported by the default mode network (DMN) (Wirth et al., 2011; St Jacques et al., 2011).

The Network Neuroscience Theory of Human Intelligence, grounded in network control theory (NCT), emphasizes a whole-brain neurobiological explanation for gF and gC (Barbey, 2018). NCT posits that transitions between brain states can be controlled by input introduced at specific regions, which then propagate (Gu et al., 2015). Specific states require less energy to reach depending on the connectivity of input regions. Modal controllability measures the efficiency with which input into a region can control the brain into hard-to-reach states requiring high energy (Gu et al., 2015). It is highest in regions with weak node strength (i.e., magnitude of connections), usually connected by long-range connectivity facilitating global integration. In contrast, average controllability measures the efficiency with which input into a region can control the brain into easy-to-reach states requiring low energy (Gu et al., 2015). It is highest in regions with high node strength, usually connected by short-range connections enabling quick signaling. Because gF involves assembling novel brain representations, a high-effort process presumably demanding high energy, modal controllability may underlie gF (Barbey, 2018). Consistent with this, high modal controllability regions are concentrated in FPN and CON (Gu et al., 2015), networks associated with gF. As gC involves accessing well-established representations, a low-effort process presumably requiring low energy, average controllability may underlie gC (Barbey, 2018). In line with this, high average controllability regions are concentrated in the DMN, a network associated with gC (Gu et al., 2015). This also implies g may relate to controllability (Barbey, 2018; Anderson et al., 2023; Wilcox et al., 2026). g may arise from the capacity to flexibly transition between network states, enabled by network controllers that move the system into both easy-to-reach (average controllability) and hard-to-reach (modal controllability) states. Prior studies have linked g to controllability (Lee et al., 2020; Anderson et al., 2023; Wilcox et al., 2026), but have yet to compare this relationship to those with gF and gC.

Average controllability, modal controllability, and node strength (i.e., sum of connection weights for a region) are mathematically related. Recent work has demonstrated that, under the current standard formulation, average controllability is approximately equivalent to the second degree (i.e., sum of squared connection weights for a region), while modal controllability is exactly equivalent to the second degree (Rubinov, 2025). Notably, average controllability can be made arbitrarily close to the second degree by tuning a free normalization parameter. While these metrics have been observed to exhibit meaningful differences (Tang et al., 2017; Tu et al., 2018; Cornblath et al., 2019; Parkes et al., 2021; Deng et al., 2022; He et al., 2022), their tight mathematical relationships raise a concern. As currently formulated, they may be ill-suited to serve as distinct neurobiological characteristics underlying separate cognitive domains that the Network Neuroscience Theory of Human Intelligence (Barbey, 2018) proposes. However, this has yet to be empirically established.

Control theory has traditionally been applied to structural connectivity (SC) (Karrer et al., 2020). However, recent work suggests alternative network models may better capture significant features of brain connectivity (Shah et al., 2019; Cai et al., 2021; Zhang et al., 2022; Menon and D’Esposito, 2022; Deng et al., 2022; Wang et al., 2025; Papallo et al., 2025), such as dynamic functional connectivity (dFC), where FC refers to statistical coupling in brain region activity. We use the term dFC in this article to refer to FC measured at multiple time points within a single run—this might be more accurately labelled time-varying FC, given ongoing debate on the dynamicity of FC measured this way (Lurie et al., 2020; Liégeois et al., 2017). Deng et al. (2022) demonstrated that evolving connectivity reduced the energy required to transition between brain activity states, highlighting the importance of temporal variance observed in FC (Preti et al., 2017). Papallo et al. (2025) also investigated average and modal controllability in a time-variant NCT model using dFC, under the assumption that stimulation of one region could influence activity in another region to an extent proportional to their baseline FC. However, neither analyses systematically examine the relationships between average controllability, modal controllability, and node strength with the cognitive domains of interest to this study. Here, we also propose a complementary approach. Because dFC patterns recur (Nomi et al., 2017), we suggest that clustering can be used to identify dominant, recurring connectivity regimes under which network control can be examined. Controllability computed within each state estimates a region’s control capacity under the connectivity architecture defined by the regime. These network states could be examined during rest as an initial exploration, on the theoretical grounds that rest represents a free sampling of the repertoire of connectivity configurations that task demands constrain (Deco and Jirsa, 2012).

This study aims to advance our understanding of the relationship between core cognitive domains and controllability. Note, however, that our study design does not test the critical hypothesized link between control energy and cognitive effort—rather, our aim is to directly examine the relationship between control energy metrics and cognitive domains. Specifically, we investigate whether standard implementations of average controllability, modal controllability, and node strength measured in SC and dFC states predict g, gF, and gC, extending prior work on network control in dFC (Deng et al., 2022; Papallo et al., 2025). We further identify brain regions contributing to successful prediction and examine these regions in relation to current models linking brain networks to cognitive functioning (Jung and Haier, 2007; Duncan, 2010; Margulies et al., 2016). First, we hypothesized that dFC-derived metrics would outperform SC in predicting cognitive performance. Second, we hypothesized that modal controllability would exhibit a stronger relationship with gF, whereas average controllability will show a stronger relationship with gC (Barbey, 2018). We further predicted that high average controllability regions will be more influential in predicting gC, and high modal controllability regions will be more influential in predicting gF.

## 2 Methods

### 2.1 Participants

Data were obtained from the HCP1200 release, which received ethics approval from the Washington University Institutional Review Board (Smith et al., 2013). Our prior analysis included 950 healthy participants with complete 3 T resting-state fMRI scans, cognitive scores of interest, and relative framewise displacement (FD) < 0.2 mm (Ng et al., 2024). For the present study, we removed five participants without diffusion MRI data. Sample characteristics are reported in Table 1.

**Table 1:** Sample descriptive statistics.

Sample characteristics	Statistics
Age	28.64 ± 3.71 [22 to 37]
Years of education	14.99 ± 1.75 [11 to 17]
Handedness (Right + / Left -)	66.33 ± 43.94 [−100 to 100]
Gender	
Male	47.09% [450]
Female	52.91% [500]
Race	
White	75.98% [718]
Black/African American	13.23% [125]
Asian/Native Hawaiian/Other Pacific Islander	6.35% [60]
American Indian/Alaskan Native	0.21% [2]
More Than One	2.43% [23]
Unknown or Not Reported	1.80% [17]
g	0.36 ± 3.15 [−10.31 to 8.91]
gF	17.10 ± 4.70 [5 to 24]
gC	117.14 ± 9.37 [90.69 to 148.54]

For continuous variables, the mean, standard deviation, and range are reported. For discrete variables, the percentage and count are reported.

### 2.2 Cognitive domains

To estimate g, we conducted a bifactor confirmatory factor analysis (CFA; Dubois et al., 2018) on all 1,192 participants with complete cognitive test scores using 10 tests (Table 2) from the NIH Toolbox (http://www.nihtoolbox.org) and Penn Computerized Neurocognitive Battery (Gur et al., 2010). The CFA factor structure can be seen in Figure 1. Model fit metrics were assessed.

**Table 2:** Cognitive measures.

Measure	Assesses
Dimensional change card sort (CardSort)	Cognitive flexibility
Flanker inhibitory control and attention (Flanker)	Cognitive control
List sorting working memory (ListSort)	Working memory
Picture sequence memory (PicSeq)	Visual episodic memory
Picture vocabulary (PicVoc)	Picture-based vocabulary
Pattern comparison processing speed (ProcSpeed)	Simple processing speed
Oral reading recognition (ReadVoc)	Reading-based vocabulary
Penn progressive matrices (PMAT24)	Fluid ability
Penn word memory (WordMem)	Verbal episodic memory
Penn line orientation (LineOrient)	Spatial orientation

**Figure 1:**
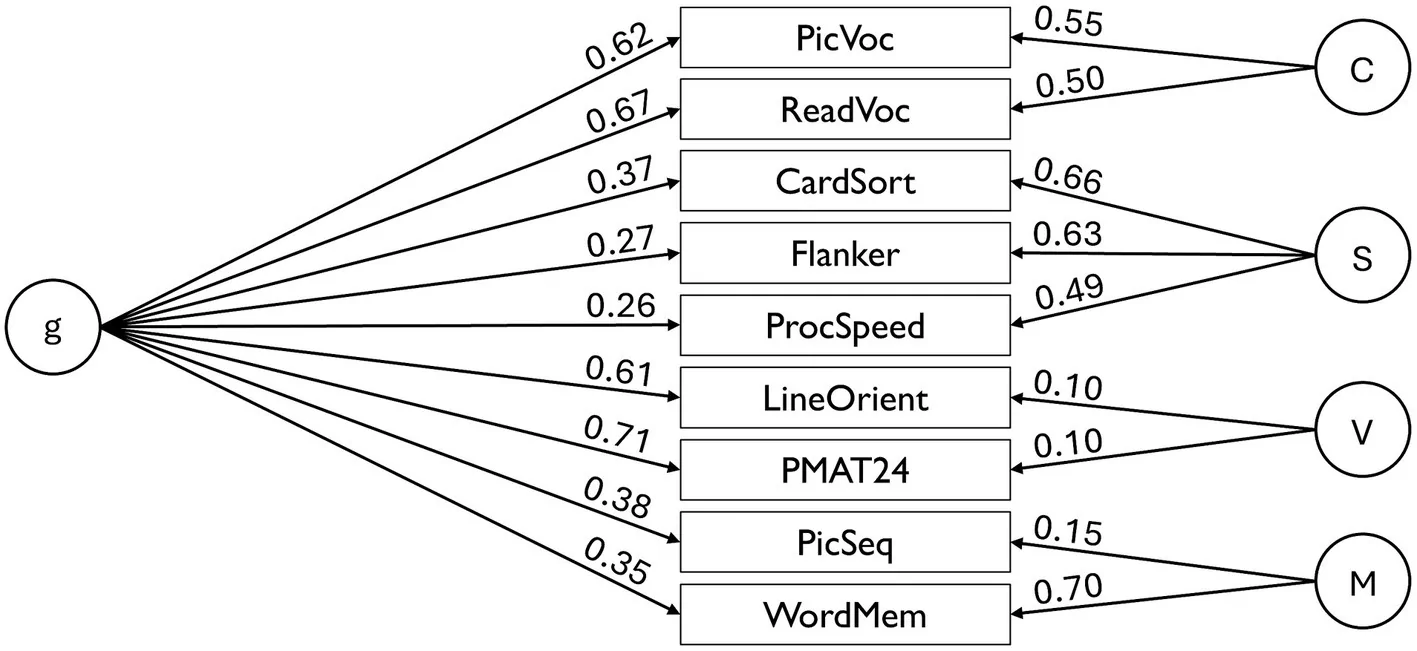
Confirmatory factor analysis for *g*. CFA was conducted with the same factor structure as specified by Dubois et al. (2018). The factor C is “cry,” S is “spd,” V is “vis,” and M is “mem.” Consistent with their CFA, ListSort was omitted for convergence. ListSort was included in other estimates of *g* described in Supplementary Section 1.

gF and gC were operationalized as Penn Progressive Matrices and Picture Vocabulary Test scores, respectively, as per prior literature (Hearne et al., 2016; Greene et al., 2018; Tadayon et al., 2020). Additional rationale is provided in Supplementary Section 1. We assessed alternative operationalizations post-hoc (e.g., g from Principal Component Analysis and exploratory factor analysis, gF and gC as averages across tests the NIH Toolbox assigns to each domain).

### 2.3 fMRI collection and preprocessing

Details can be found in the original publications (Smith et al., 2013; Salimi-Khorshidi et al., 2014). Briefly, 3 T resting-state fMRI data were collected over two sessions of two 15-min runs in left–right and right–left phase-encoding directions using a Siemens “Skyra” scanner with a multiband slice acquisition sequence (TR = 720 ms, TE = 33 ms, flip angle = 52^o^, voxel size = 2 mm isotropic, 72 slices, multiband acceleration factor = 8, 104 × 90 matrix). The surface-based data underwent minimal preprocessing (B0 distortion correction, co-registration to T1-weighted images, normalization to the surface template), ICA-FIX, and regression with 24 motion parameters (three translations and three rotations, their temporal derivatives, and their squares) (Satterthwaite et al., 2013). Voxel time series were demeaned and averaged in 360 HCP-MMP1.0 atlas cortical regions (Glasser et al., 2016).

### 2.4 Dynamic functional connectivity

dFC states were identified using Leading Eigenvector Dynamic Analysis (LEiDA; Preti et al., 2017; Cabral et al., 2017). Unlike the traditional sliding-window approach used in prior dFC network control analyses (Deng et al., 2022; Papallo et al., 2025), LEiDA computes dFC(t) at each time point, thereby eliminating window-length dependencies and reducing temporal autocorrelation between successive dFC matrices (Cabral et al., 2017; Lord et al., 2019; Preti et al., 2017). The phase θ(n,t) for each n region at each time point t was derived using the Hilbert transform. This transform was applied separately to each run. For any two regions n and m, BOLD signal synchronization was quantified as FC(n,m,t) = cos(θ(n,t) - θ(m,t)). cos(0^o^) = 1 indicates perfect coherence, cos(90^o^) = 0 indicates an orthogonal relationship, and cos(180^o^) = −1 indicates perfect anti-coherence. Eigen decomposition was performed on dFC(t) (N x N) at each t to reduce its dimensionality to the leading eigenvector, LE(t) (N x 1), to facilitate clustering (e.g., reducing computational cost).

k-medians clustering with Manhattan distance was applied to LE(t) pooled across participants, with 500 repetitions using random initializations to reduce the risk of convergence on a suboptimal solution (Allen et al., 2014; Aggarwal et al., 2001). dFC states were then characterized as the median dFC(t) within each LE(t) cluster for each participant. The optimal number of clusters depends on the neuroscientific question under investigation (Lord et al., 2019). In our previous work (Ng et al., 2024), the elbow method indicated *k* = 4–5, consistent with values reported in similar studies. We selected *k* = 6, close to this conventional range, because it also yielded a state that delineates the FPN, a network with a well-established relationship to general cognitive ability (Jung and Haier, 2007; Duncan, 2010).

### 2.5 Structural connectivity

Connectivity is typically measured using SC in NCT analyses, where inter-regional relationships are represented by streamline counts. We utilized publicly available probabilistic diffusion MRI tractography data (Rosen and Halgren, 2021). In brief, diffusion MRI data were preprocessed and corrected for eddy currents and head motion using FSL eddy (Andersson and Sotiropoulos, 2016). The HCP-MMP1.0 parcellation was projected from grayordinate space onto each participant’s white matter surface and converted to volumetric seed and target masks at the white/grey matter boundary. Probabilistic tractography was performed using FSL’s probtrackx2 in voxel-by-parcel mode, estimating streamline counts between each parcel pair (0.5 voxel step length, 5,000 samples, 2,000 steps, 90° curvature threshold). Within-parcel connectivity was not assessed.

To address differences in parcel size, streamline counts were normalized using fractional scaling. This approach calculates the ratio of streamlines originating from Region A and ending at Region B, to the total streamlines originating from Region A and total streamlines terminating at Region B, excluding intra-parcel streamlines.

### 2.6 Static functional connectivity

To assess whether differences between dFC and SC might be more attributable to temporal variance or the difference between structural and functional connectivity, we assessed static functional connectivity (sFC) as an intermediate modality. sFC can be considered a frequency-weighted average of dFC states capturing overall connectivity in the functional modality (Cabral et al., 2017; Ng et al., 2024).

For each participant, regional fMRI time series were concatenated across runs, Pearson’s correlations were calculated between each pair of timeseries, and resulting values were Fisher’s Z-transformed.

### 2.7 Thresholding

Probabilistic tractography generates dense matrices with false positives. Similarly, dFC and sFC are dense. NCT studies commonly utilize sparse matrices from deterministic tractography (Gu et al., 2015; Lee et al., 2020; Parkes et al., 2021) or probabilistic tractography with thresholding (Tang et al., 2017). In the main analysis, we retained 50% of connection weights with the lowest variability across participants (Roberts et al., 2017). To ensure differences were not solely attributable to thresholding, we applied this procedure to all connectomes. For detailed rationale, see Supplementary Section 3. Alternative thresholds (e.g., including the minimum spanning tree to connect all regions) were explored post-hoc.

### 2.8 Strength

We measured strength as the sum of the magnitudes of all connections to a region. Because negative FC values exist, we used the absolute sum. Summing raw signed values would allow positive and negative connections to cancel out, underestimating the strength of regions with strong connections of both signs.

### 2.9 Global controllability

For a detailed explanation of NCT metrics, refer to Supplementary Section 4. The assessment of global controllability (i.e., input into the region can drive any arbitrary state into any other state) using the controllability Gramian is a prerequisite for calculating average and modal controllability (Gu et al., 2015; Wilmskoetter et al., 2022). Regions failing this criterion were excluded. These were generally disconnected regions.

### 2.10 Average controllability and modal controllability

Average controllability measures a region’s capacity to steer brain activity toward states requiring low energy to reach. It is calculated as the trace of the controllability Gramian, which encodes the system’s low-energy impulse response. Modal controllability measures the capacity to drive activity towards states that require high energy. It is measured as the sum of the region’s eigenvector entries (control capacity) weighted towards smaller eigenvalues (difficult-to-control modes) of the connectivity matrix encoding the transformation between brain activity states. As average and modal controllability exhibit high correspondence with strength, we assessed Pearson’s correlations between the metrics and compared their predictive performance. Notably, when averaged across participants, absolute strength correlated with average and modal controllability at *r* > 0.9 across FC states, confirming that the strength-controllability relationship holds when strength is computed using absolute values.

Notably, fractional scaling restricted the range of controllability values relative to raw streamline counts. To address this, we investigated raw streamline counts post-hoc.

### 2.11 Brain maps

To contextualize our findings, we investigated the relationship between regions highlighted in our findings and established brain maps.

We examined the principal cortical gradient (Margulies et al., 2016), a score for each region that marks the gradual change in connectivity from transmodal (i.e., many different tasks) association to unimodal (i.e., specific functions) sensorimotor regions (Figure 2, top right). This map has been related to controllability (Parkes et al., 2021) and cognition (Yu et al., 2024). We derived the principal gradient using sFC, following Parkes et al. (2021). sFC was normalized using Fisher’s r-to-z transform and averaged across participants. The DiffusionMaps approach with the normalized_angle kernel from the BrainSpace toolbox (Vos de Wael et al., 2020) was applied.

**Figure 2:**
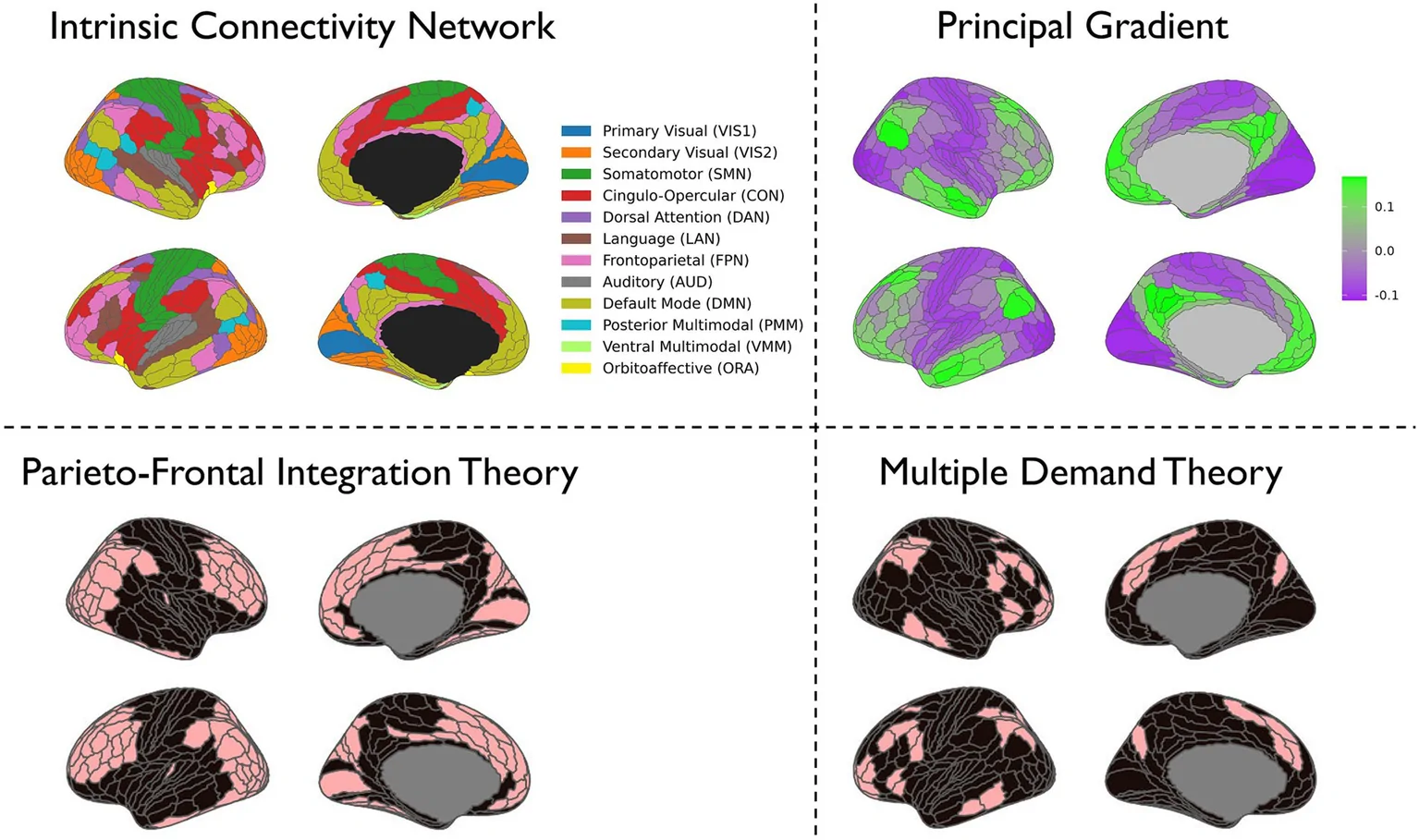
ICN, principal gradient, PFIT, and MD regions. For the principal gradient, green regions refer to transmodal association regions, and purple regions refer to unimodal sensorimotor regions. For the PFIT and MD regions, pink regions refer to the regions specified in the theories.

As we examined g, we also investigated regions thought to underlie g. These include Parieto-Frontal Integration Theory (PFIT) parcels (Figure 2, bottom left) outlined by Genç et al. (2023), and Multiple Demand Theory (MD) parcels (Figure 2, bottom right) from the extended MD system outlined by Assem et al. (2020).

Regions were also assigned to intrinsic connectivity networks (ICNs; Figure 2, top left) using the Cole-Anticevic Brain-Wide Network Partition (CAB-NP; Ji et al., 2019), which classified HCP-MMP1.0 parcels into 12 networks via community detection applied to resting-state sFC.

### 2.12 Statistical analysis

#### 2.12.1 Model prediction

Our objective was to predict cognitive measures (g, gF, and gC) using controllability metrics derived from different modalities (SC, sFC, and dFC). We employed kernel ridge regression because of its robustness to collinearity and its ability to partition explained variance across predictor subsets (Chen et al., 2022; Deng et al., 2022). Kernel ridge regression extends ordinary ridge regression through the kernel function, enabling the capture of nonlinear relationships. Consistent with Deng et al. (2022), we used the cosine kernel. Separate kernels were constructed for controllability metrics derived from each network modality, and kernel weightings were used to attribute explained variance to each network modality. Separate kernels were also constructed to assess the explained variance of each connectivity matrix. Methodological details are described in Dupré la Tour et al. (2022).

We employed repeated 10-fold nested cross-validation. Participants were randomly divided into 10 folds, ensuring related individuals were kept within the same fold (Ooi et al., 2022). In each iteration, nine folds were used for training, and 5-fold cross-validation was used to optimize regularization hyperparameters. Age and self-reported binary gender (sex was not recorded) were regressed from brain and behavior variables to control for effects without compromising sample size and generalizability (Agelink van Rentergem et al., 2020). We also conducted gender-restricted analyses post-hoc. To avoid test set contamination, age and gender coefficients, and the mean and standard deviation, were computed from each training set and applied to the test set for confounder regression and standardization. For each test set, we computed total variance explained using the coefficient of determination (*R*^2^; ranges from -∞ to 1, 1 indicates perfect prediction) and subset contributions. Notably, *R*^2^ is not calculated as the square of Pearson’s correlation (it is only equivalent in simple linear regression). Zero means in the test set are required for score decomposition, but this risks overoptimistic results. Therefore, we report *R*^2^ without demeaning and report subset contributions as percentages of the *R*^2^ after demeaning. To mitigate bias in participant assignment, the procedure was repeated five times, and scores were averaged.

Statistical significance of *R*^2^ was assessed using permutation. We permuted cognitive scores 1,000 times, permuting within blocks of unrelated participants (Ooi et al., 2022).

#### 2.12.2 Model comparison

Since a permutation test is inappropriate (Chen et al., 2022; Nadeau et al., 1999), we used the corrected resampled *t*-test to compare *R*^2^. Importantly, cross-validation directly penalizes overfitting (e.g., from excess predictors), enabling fairer comparisons across models of differing complexity (Hastie et al., 2017). Similar to Wilcox et al. (2026), we observed that combining redundant predictors did not improve *R*^2^, and adding non-informative predictors could even reduce *R*^2^. FDR correction was applied across statistical tests conducted on 108 tests. These include 27 *R*^2^ scores (3 cognitive variables × 3 network metrics × 3 modality combinations) and 81 *R*^2^ comparisons (6 × 3 dFC vs. SC, 6 × 3 controllability vs. strength, 6 × 3 cognitive vs. cognitive, 9 × 3 sFC vs. SC and dFC).

#### 2.12.3 Regional importance to prediction

We employed the Haufe approach to assess regional importance to the prediction (Haufe et al., 2014; Chen et al., 2022). Regional importance was calculated as the covariance between each predictor and the predicted cognitive scores within the training set, averaged across iterations. Positive values indicate that higher controllability predicts higher cognitive scores, whereas negative values indicate the opposite. Statistical significance was assessed using 1,000 permutations. FDR correction was applied across SC and dFC models of interest (i.e., 360 regions × 7 matrices × 3 cognitive metrics × 3 network metrics = 22,680 tests).

#### 2.12.4 Correspondence between brain maps and regional importance

We examined the correspondence between regional importance and brain maps.

For continuous maps (controllability and the principal gradient), we employed quadratic regression to assess quadratic model fit and Spearman’s correlation to assess the monotonic direction to examine whether regions where higher controllability predicted better cognitive performance were associated with higher values on the continuous map, and vice versa. We measured R^2^ for the quadratic regression and its statistical significance using spin permutation (to account for spatial autocorrelation) with 1,000 spins (Alexander-Bloch et al., 2018). We also measured Spearman’s correlations and their statistical significance using spin permutation with 1,000 spins.

For binary maps (PFIT and MD regions), we quantified overlap with regions showing significant regional importance using the Dice coefficient, adjusted for class size (0 = no overlap; 1 = perfect overlap). Statistical significance was assessed using permutation (Cookson and D’Esposito, 2023).

FDR correction was applied across statistical tests conducted on 154 tests, including 56 quadratic regression *R*^2^ (3 network metrics × 7 connectivity matrices for network metric vs. regional importance for g, 1 network metric × 7 connectivity matrices for network metric vs. regional importance repeated for gF and gC, 3 network metrics × 7 connectivity matrices for principal gradient vs. regional importance), 56 Spearman’s correlations (same as quadratic regression), and 42 Dice coefficients (2 binary maps × 3 network metrics × 7 connectivity matrices).

See Figure 3 for an analysis flowchart.

**Figure 3:**
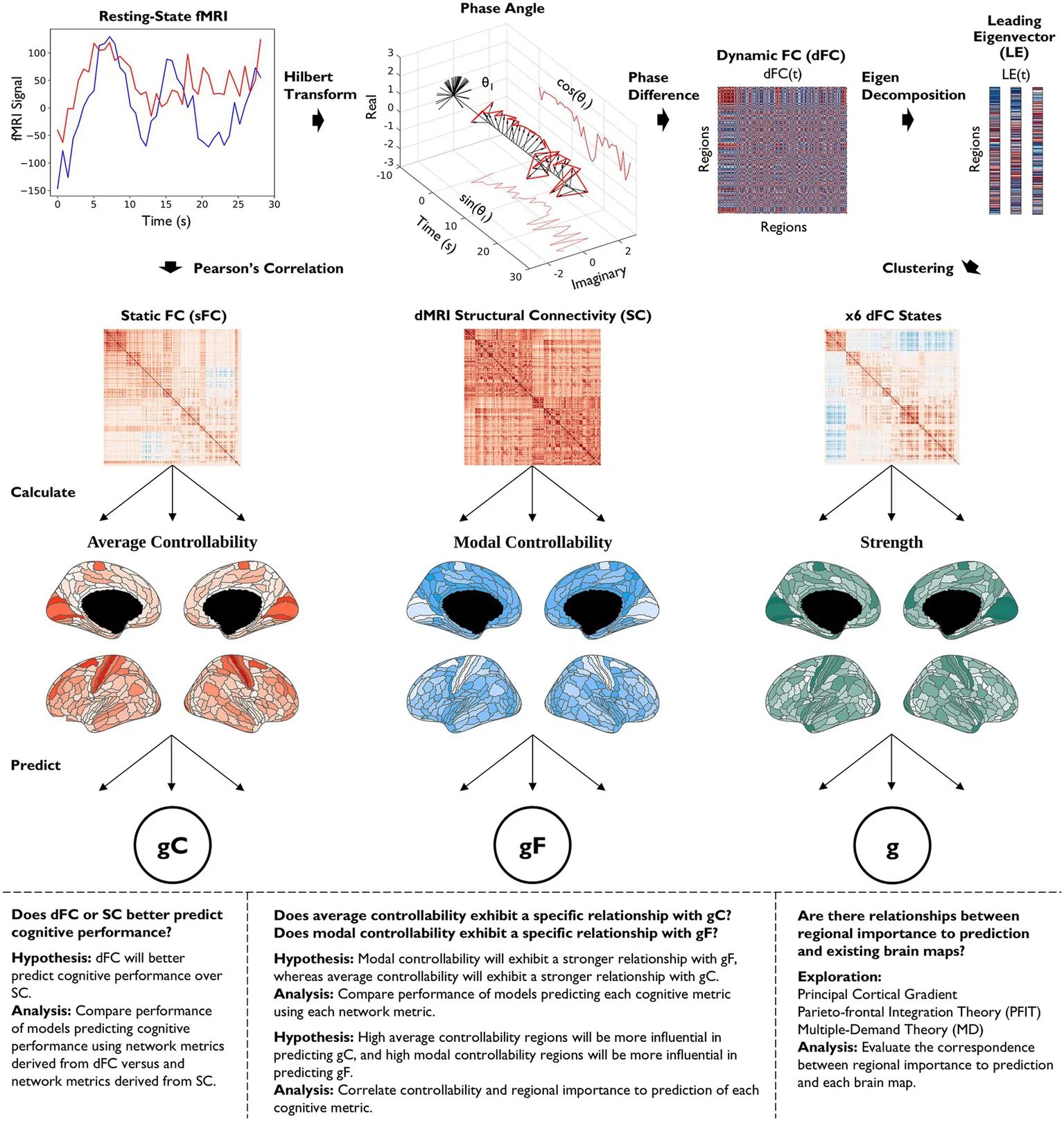
Analysis flowchart. Eight connectivity matrices were generated for each participant. Dynamic functional connectivity (dFC) states were derived from resting-state fMRI. For each region’s fMRI timeseries, a Hilbert transform was applied to obtain the instantaneous phase at each time point, and dFC was quantified as the phase difference between each pair of regions, yielding a *dFC*(*t*) matrix per time point. Eigen decomposition was applied to each *dFC*(*t*) matrix to obtain its leading eigenvector, *LE*(*t*), facilitating clustering of the matrices. K-medians clustering was used to cluster *LE*(*t*) across time points and participants. The average *dFC*(*t*) within each cluster was computed for each participant, yielding six dFC states per participant. Static functional connectivity (sFC) was calculated as the Pearson’s correlation between each pair of regions’ fMRI timeseries. Structural connectivity (SC) was derived from dMRI via diffusion tractography. Average controllability, modal controllability, and strength were calculated as regional scores from each of these eight connectivity matrices. Combinations of these regional scores were then used to predict *g*, *gF*, and *gC*. These analyses addressed three questions: (1) whether dFC or SC better predicts cognitive performance, (2) whether average and modal controllability show distinct relationships with *gC* and *gF* respectively, and (3) whether regional importance to prediction corresponds to existing brain maps.

## 3 Results

### 3.1 Cognitive domains

We investigated g using factor analysis, assessed using the comparative fit index (CFI; good fit > 0.95), root mean squared error of approximation (RMSEA; good fit < 0.06), standardized root mean squared residual (SRMR; good fit < 0.08), and Bayesian information criterion (BIC; lower values are better). The model demonstrated good fit: CFI = 0.97, RMSEA = 0.052, SRMR = 0.032, and BIC = 73,014. g correlated highly with gF (*r* = 0.81) and gC (*r* = 0.70), while gF and gC were moderately correlated (*r* = 0.44).

### 3.2 Network matrices and controllability

We investigated SC, sFC, and six dFC states shown in Figure 4. Consistent with prior studies (Rosen and Halgren, 2021), SC exhibited lower interhemispheric connectivity and weak correspondence (*r* = 0.25; see Supplementary Table S1 for all correlations) with sFC-derived intrinsic connectivity networks (ICNs). sFC most closely resembled State 1 (*r* = 0.87), which is expected given that this state occurs most frequently—it likely serves as a default “meta-stable” state to which the system returns (Cabral et al., 2017; Vohryzek et al., 2020; Ng et al., 2024).

**Figure 4:**
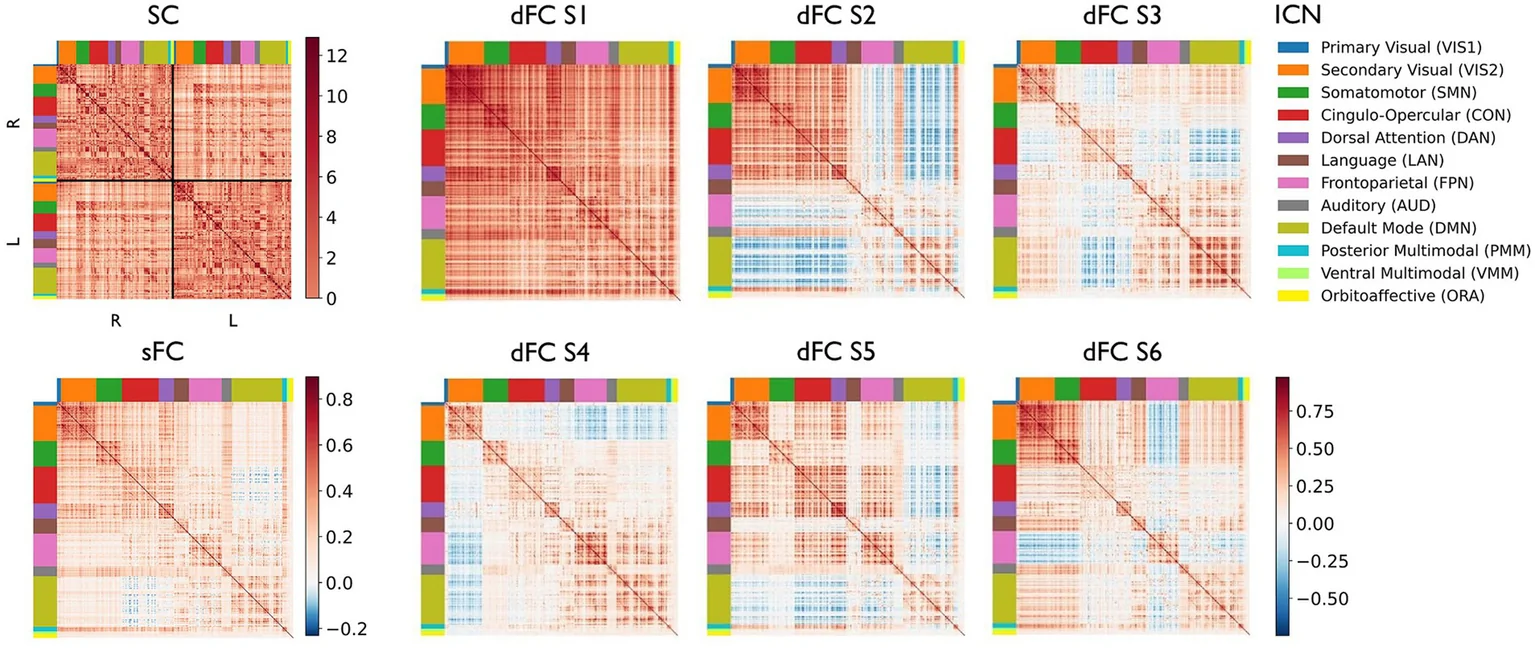
Network matrices. SC refers to structural connectivity, sFC refers to static functional connectivity, dFC refers to dynamic functional connectivity, each S# refers to one of 6 dFC states, and ICN refers to intrinsic connectivity networks. The visualized SC and sFC matrices were constructed by finding the median across participant SC and sFC matrices, respectively. The visualized dFC matrices were constructed by finding the median across the participant dFC state matrices, where participant dFC state matrices were constructed as the median across dFC(t) classified to each state for each participant. The SC matrix was sorted by both ICN and hemisphere (R for right, L for left) to highlight the difference between within- and between- hemispheric connectivity, as there was not much correspondence with ICNs. The sFC and dFC matrices were sorted by ICN only, as there was little difference between hemispheres.

Regions with high average controllability generally differed between the eight matrices and corresponded to regions of high strength (Figure 5). For SC, these were sensory and motor regions and parietal, lateral temporal, and dorsolateral prefrontal regions within the DMN, consistent with prior SC studies (Gu et al., 2015; Lee et al., 2020). State 1 and sFC showed some overlap with SC, while States 2–6 were more distinct. Unlike State 1, which was characterized by a single coherent group of networks, States 2–6 each comprised two groups of networks showing positive coherence within each group and anticoherence between groups. Networks with high strength in the state also contained regions of high average controllability. State 2 highlighted secondary visual network (VIS2), CON, and somatomotor network (SMN) regions, characterizing anticoherence between VIS2/CON/SMN and FPN/DMN. State 3 highlighted DMN regions, characterizing anticoherence between DMN and CON. State 4 highlighted FPN, DMN, and VIS2 regions, characterizing anticoherence between FPN/DMN and VIS2. State 5 highlighted CON and dorsal attention network (DAN) regions, characterizing anticoherence between CON/DAN/FPN/VIS1/VIS2 and DMN. State 6 highlighted VIS2 and SMN regions, characterizing anticoherence between VIS2/SMN and FPN.

**Figure 5:**
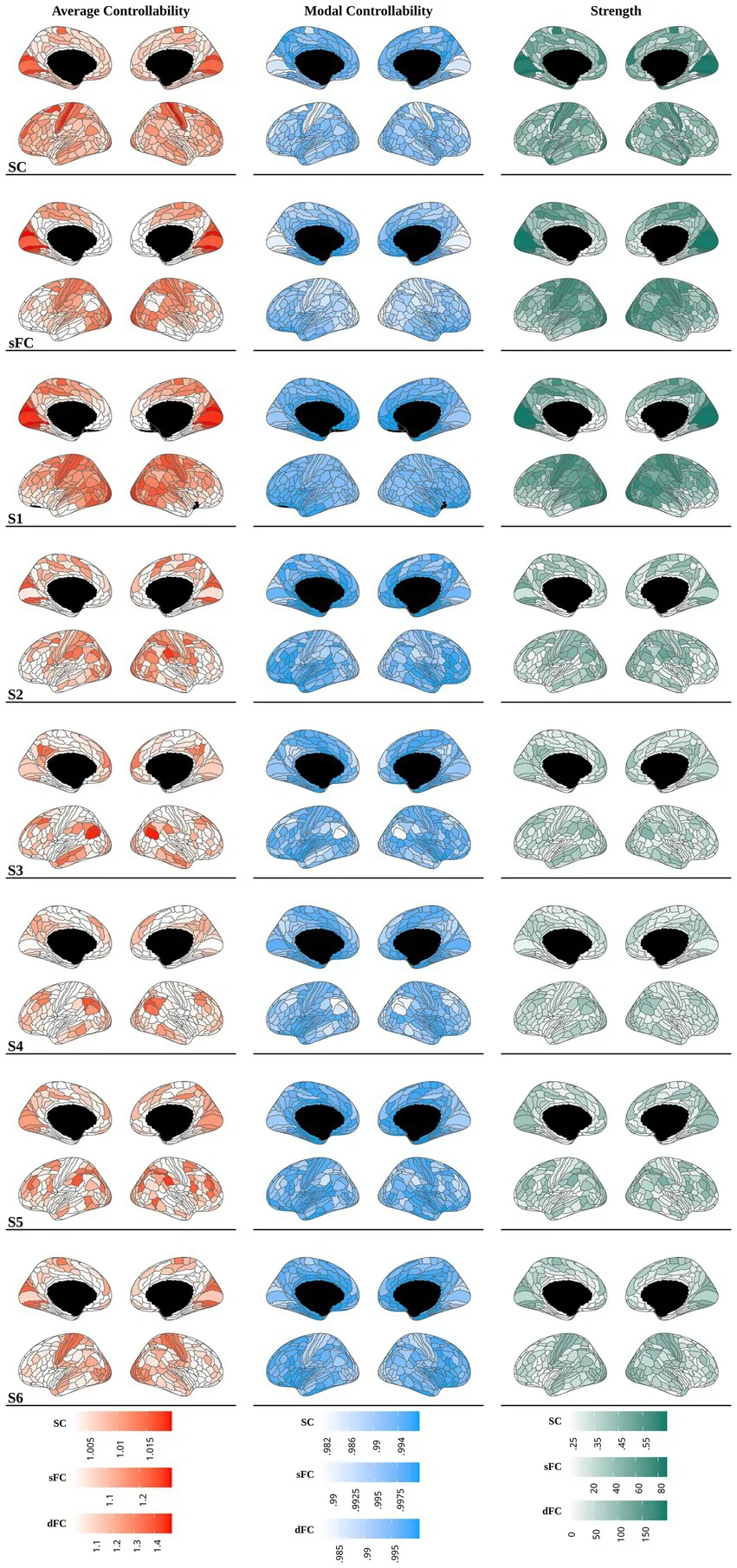
Brain controllability and strength. S# refers to one of 6 dFC states. Separate color scales were used for each modality to enhance visibility.

Regions with the highest modal controllability were more consistent across the eight matrices and corresponded to regions with low strength. These included orbitofrontal, inferior frontal opercular, superior and inferior lateral temporal, medial temporal, and cingulate regions of the DMN, FPN, and CON. SC observations also align with prior SC studies (Gu et al., 2015; Lee et al., 2020).

Average controllability and modal controllability correspond closely with strength on average (Figure 6), but because predictive models rely on interindividual variation, these metrics may still carry distinct information. For each connectivity matrix, we also computed these correlations separately within each region, across participants. While the group-average correlation was high, per-region correlations were generally moderate, suggesting these metrics may still be worth analyzing separately.

**Figure 6:**
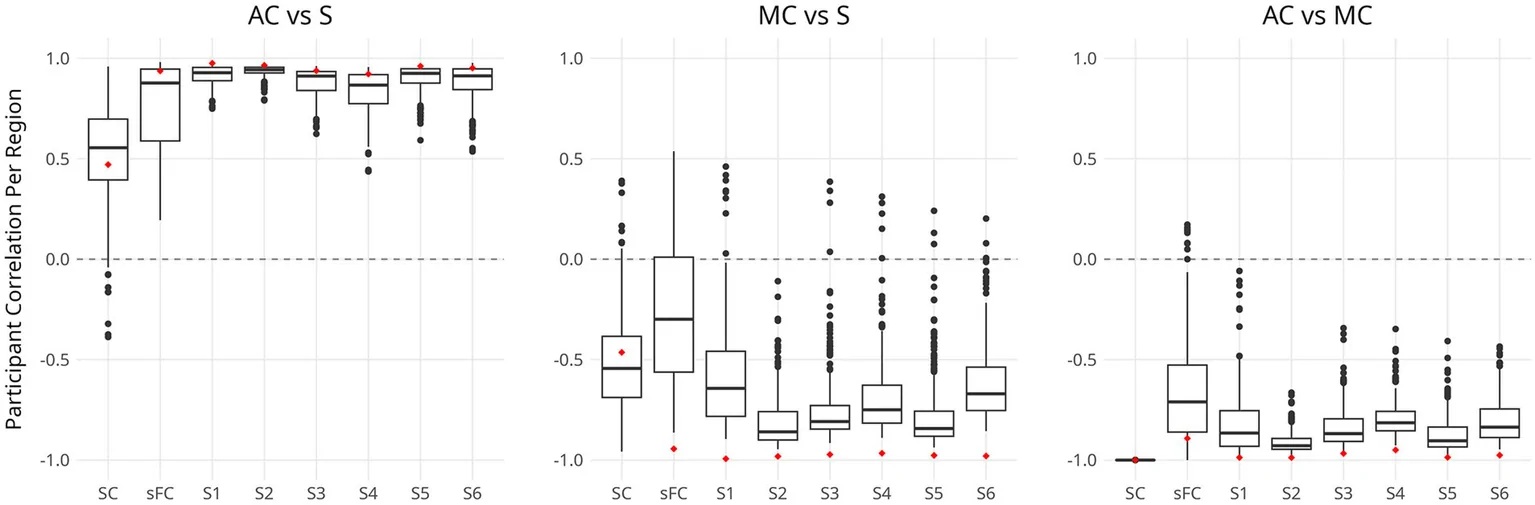
Correspondence between average controllability, modal controllability, and strength. AC, MC, and S refer to average controllability, modal controllability, and strength, respectively. Each S# refers to a dFC state. Each observation in the box plots is a Pearson’s correlation between participant values for each region. Each red point characterizes the Pearson’s correlation between the group-average values across regions, for comparison.

### 3.3 dFC best predicts cognitive performance

Figure 7 presents the *R*^2^ performance of models predicting g, gF, and gC using controllability and strength derived from SC and dFC. Because our hypotheses focus on SC and dFC, statistical analyses were restricted to SC and dFC models. We employed multivariate kernel ridge regression, which permits partition of variance explained by each modality. Figure 7 reports the variance contribution of each modality normalized as a percentage of the total, reflecting the optimal combination for prediction according to the algorithm. Negative values indicate the modality reduced the variance explained. Model performance was evaluated using cross-validation, and permutation testing was conducted to determine significance.

**Figure 7:**
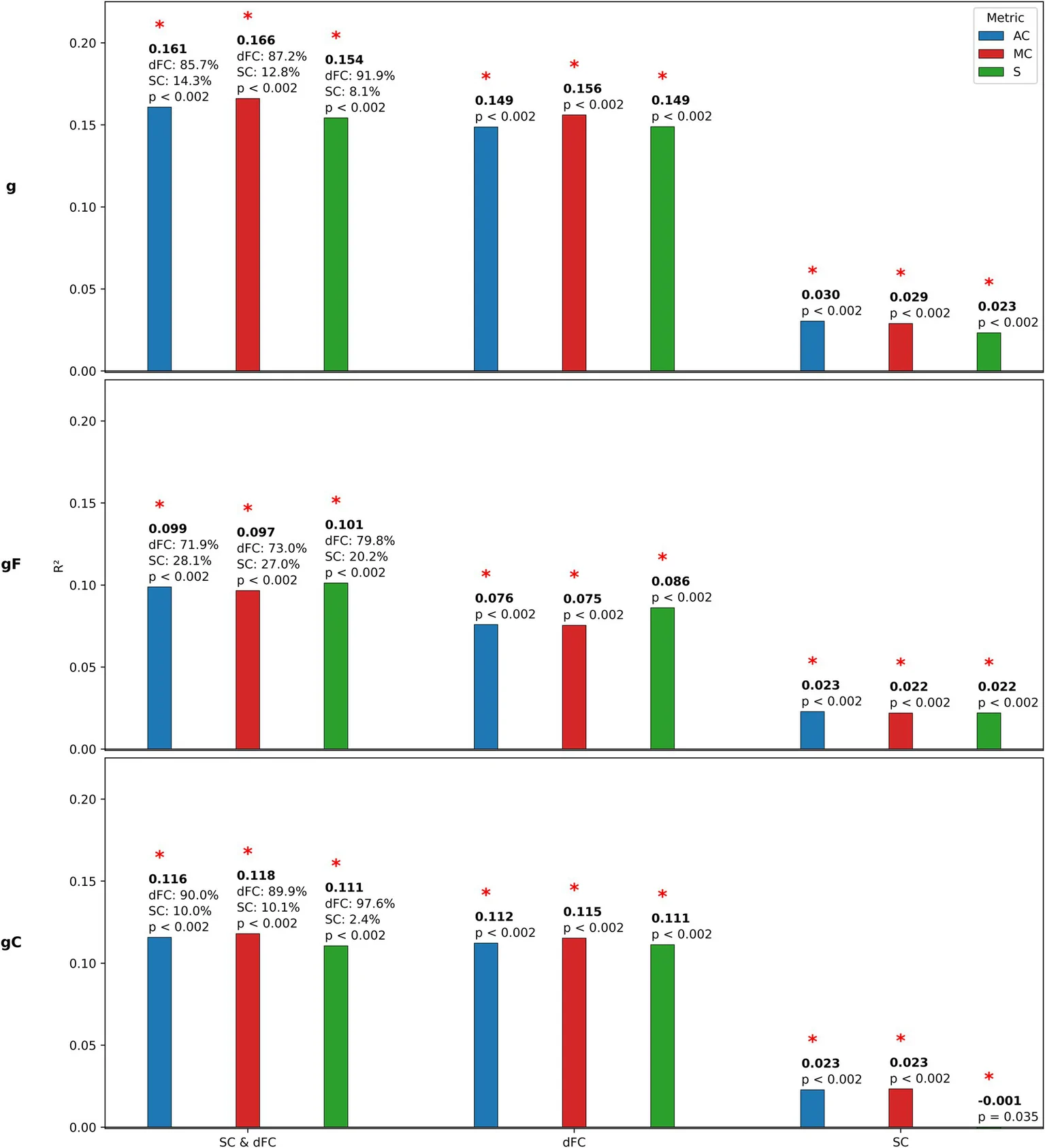
*R^2^* for predicting *g*, *gF*, and *gC* using models including SC and dFC. AC, MC, and S refer to average controllability, modal controllability, and strength, respectively. Above each bar, the *R2*, percentage contributions by each modality, and FDR-corrected *p*-values are reported. A “<” preceding a *p*-value indicates that no permuted value exceeded the true value. The red point indicates *p* < 0.05.

As expected, dFC was more predictive than SC for g, gF, and gC. Moreover, in combined models, dFC explained more variance than SC. Statistical comparisons across models indicated dFC explained significantly more variance than SC for average controllability (AC), modal controllability (MC), and strength (S) for g (AC: *R*^2^ = 0.12, *p* = 0.0021; MC: *R*^2^ = 0.13, *p* = 0.0018; S: *R*^2^ = 0.13, *p* = 0.00086) and gC (AC: *R*^2^ = 0.089, *p* = 0.016; MC: *R*^2^ = 0.092, *p* = 0.0088; S: *R*^2^ = 0.11, *p* = 0.0018), but only strength for gF (AC: *R*^2^ = 0.053, *p* = 0.087; MC: *R*^2^ = 0.053, *p* = 0.087; S: *R*^2^ = 0.064, *p* = 0.0094) (see Supplementary Table S2 for a complete table of *R*^2^ differences and *p*-values). SC and dFC models did not significantly explain more variance than dFC models for any measure. This suggests dFC explains significantly more variance than SC across most models employed, but not all, and adding SC to the dFC model does not significantly improve prediction. Notably, however, SC contributed nonzero variance when included alongside dFC and yielded an increase in *R*^2^, suggesting that SC captures information not fully redundant with dFC. The difference between SC and dFC observed was robust to variation in thresholding, number of states, SC normalization, cognitive measure, and gender restriction (Supplementary Section 5). Interestingly, though, SC models performed even worse in males. In contrast, sFC contributed minimally when included alongside dFC (Figure 8), indicating sFC provides little additional cognitively-relevant information. Furthermore, sFC was significantly less predictive than dFC for g, gF, and gC (Supplementary Section 2).

**Figure 8:**
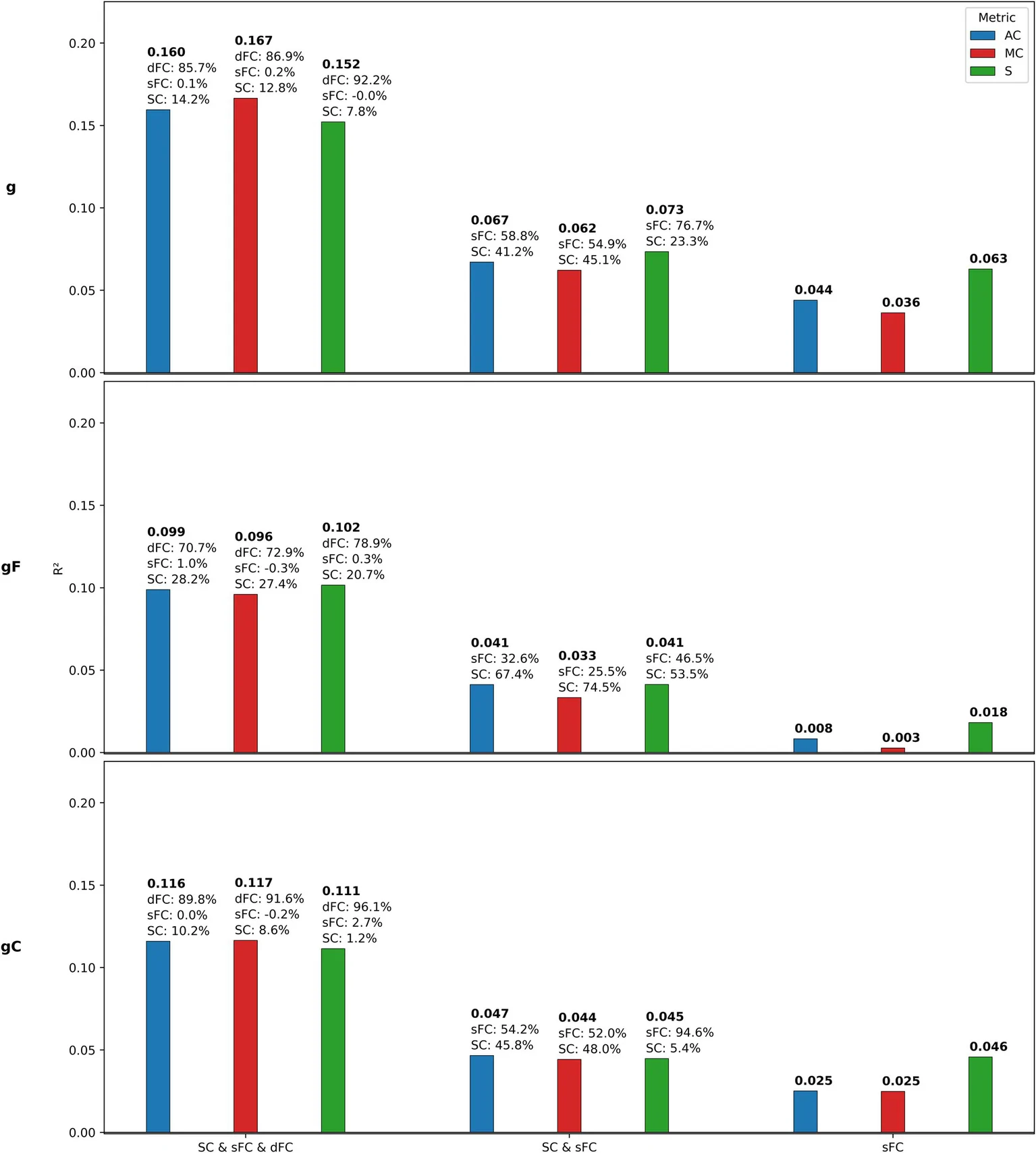
*R*^2^ for predicting *g*, *g*F, and *g*C using models including *s*FC. AC, MC, and *S* refer to average controllability, modal controllability, and strength, respectively. On top of each bar, the *R*^2^ and percentage contributions by each modality are reported. Permutation testing was not done, as these were not the main models of interest.

After establishing the variance contribution of each modality, we also examined the variance contribution of individual connectivity matrices within our main modalities of interest, SC and dFC (Supplementary Figure S1). Averaged across network metrics, for g, States 2 (21.09%) and 5 (23.90%) contributed the most variance. For gF, SC (26.06%) and State 2 (23.90%) were the largest contributors. For gC, States 5 (24.32%) and 6 (26.43%) contributed the most variance.

### 3.4 Average and modal controllability show limited specificity in predicting gF and gC

Average and modal controllability significantly predicted gF and gC (Figure 7), but exhibited nearly identical *R*^2^. Strength showed comparatively larger differences in performance, so we tested whether the magnitude of this difference was statistically significant. It was not, for all comparisons (Supplementary Table S3). The similarity between controllability and strength observed was robust to variation in thresholding, number of states, SC normalization, cognitive measure, and gender restriction for the SC and dFC model (Supplementary Section 5). Interestingly, however, the differences in predictive performance between strength and controllability in SC appeared to depend on the thresholding. At the threshold used in the main analysis, controllability and strength differed by only ~0.0064 *R*^2^ in predicting g, but at more extreme thresholds this grew to ~0.013, driven by the relatively stable performance of controllability and declining performance of strength (Supplementary Section 5).

Average and modal controllability also better predicted g compared to gF and gC (Figure 7). We found that the magnitude of this difference was significant between g and gF in dFC and SC and dFC models (Supplementary Table S4). These results may suggest that controllability and strength most strongly predict g. The better prediction for g was robust to variation in thresholding, number of states, SC normalization, cognitive measures, and gender restriction for the SC and dFC model (Supplementary Section 5). It was also robust to variation in thresholding for the SC model (Supplementary Section 5).

### 3.5 Regional importance of average and modal controllability in predicting cognitive domains shows extensive overlap

We examined regional importance scores in SC and dFC models predicting g, given their superior performance (Figure 9). Regional importance scores in individual SC and dFC models strongly correlated with those from combined SC and dFC models (*r* > 0.94), suggesting examining individual models would also be largely redundant.

**Figure 9:**
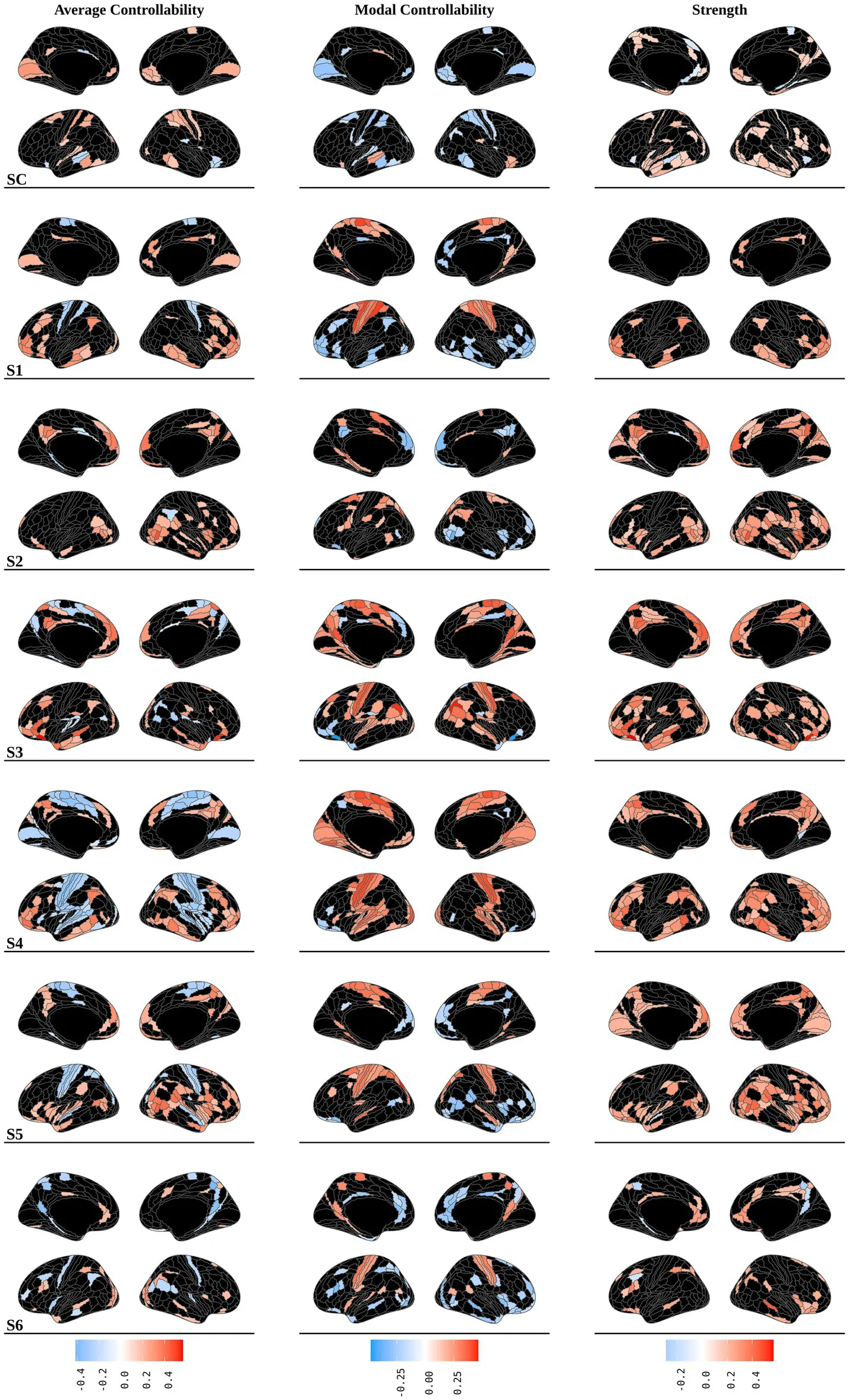
Regional importance for the prediction of g by average controllability, modal controllability, and strength in the SC and dFC model. S# refers to one of 6 dFC states. Red labels positive importance, blue labels negative importance, and black labels non-significance. For the prediction of gF and gC, see Supplementary Figures S2, S3, respectively.

Regional importance for average and modal controllability exhibited a strong negative relationship with each other across cognitive domains (*r* = −0.85), and correlated positively with strength for average controllability (*r* = 0.87) and negatively with strength for modal controllability (*r* = −0.68). This correspondence was also observed when restricted to regions with significant regional importance (Figure 9 and Supplementary Figures S2, S3). However, among significant regions, while average and modal controllability showed a roughly proportionate mix of positive and negative regional importance, strength showed predominantly positive regional importance—lower strength generally did not predict higher g.

For SC, regions where average controllability and strength positively predicted g (and modal controllability negatively predicted g) were located in somatomotor, visual, superior and inferior lateral temporal, parietal, and anterior and posterior cingulate areas. Regions where modal controllability positively predicted g (and average controllability and strength negatively predicted g) were fewer, located in orbitofrontal, opercular, middle lateral temporal, and other anterior cingulate areas. Strength additionally positively predicted g in frontal and medial temporal areas.

For dFC, significant regions showed commonality across states and generally respected sFC-based ICNs. Across states, regions where average controllability and strength positively predicted g (and modal controllability negatively predicted g) were generally located in FPN, CON, and DMN. Regions where modal controllability positively predicted g (and average controllability negatively predicted g) were generally located in primary visual (VIS1), VIS2, auditory (AUD), language (LAN), SMN, and CON.

### 3.6 Regional importance in predicting cognitive performance relates to controllability magnitude

We hypothesized regions with higher average controllability might contribute more to the prediction of gC, and regions with higher modal controllability might contribute more to the prediction of gF. This would manifest as a convex quadratic relationship without a monotonic trend—regions with high positive or negative importance should have high controllability. To test this, we calculated the quadratic regression *R*^2^ to assess the quadratic fit and Spearman’s correlation to assess the monotonic trend between regional importance and controllability for each matrix in the SC and dFC models (Figure 10).

**Figure 10:**
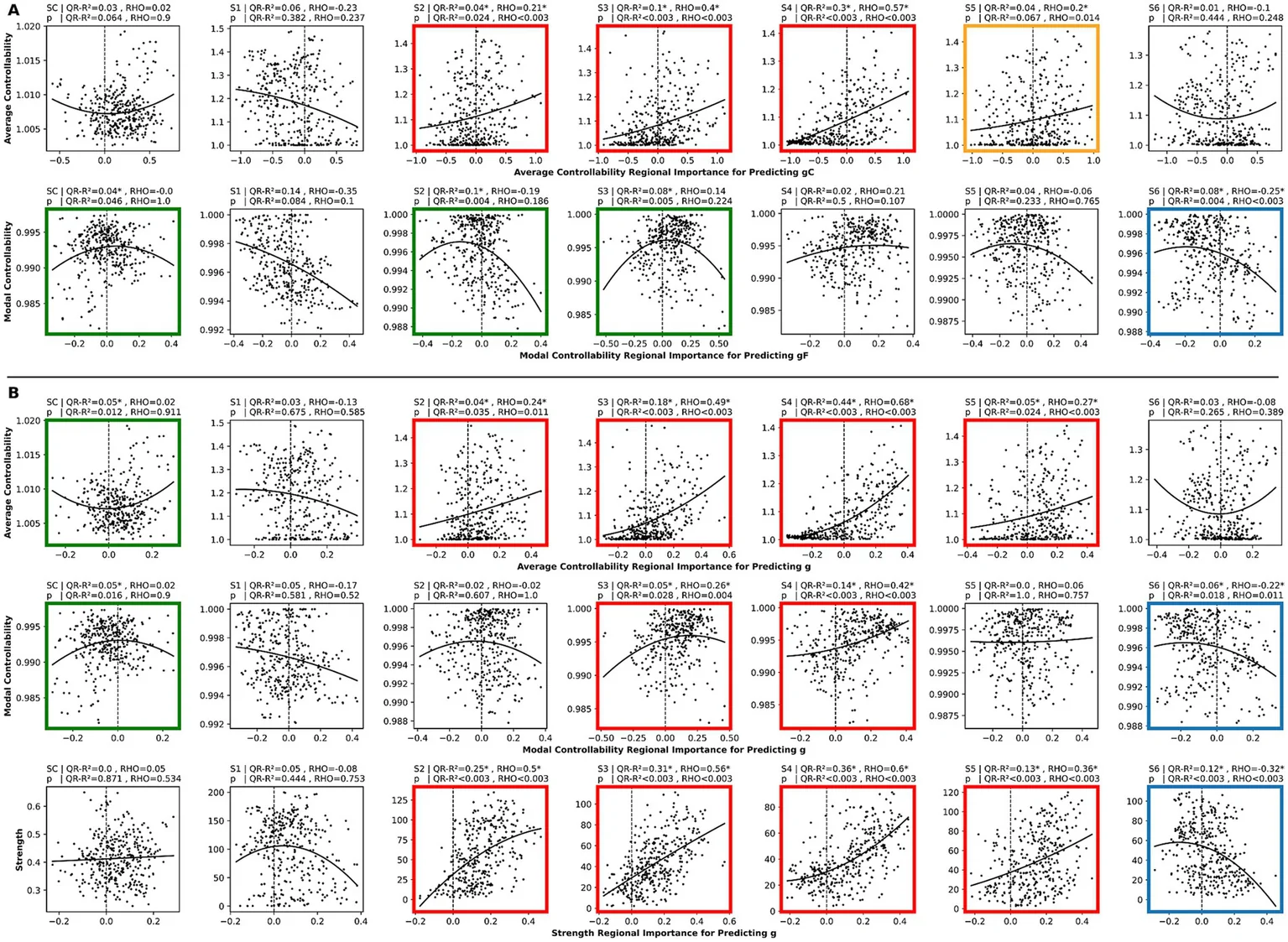
Scatterplots for the relationship between the regional importance for the prediction of *g*, *gF*, and *gC* and average controllability, modal controllability, and strength. **(A)** Relationship between average controllability and regional importance in the model predicting *gC* from average controllability, and between modal controllability and regional importance in the model predicting *gF* from modal controllability. **(B)** Relationship between each controllability metric (average controllability, modal controllability, and strength) and regional importance in the corresponding model predicting *g*. Black dots correspond to regions, dotted black lines denote the positive and negative regional importance separation, and continuous black curves represent the quadratic regression model. In the first row of the titles, QR-*R*2 refers to *R*2 for the quadratic regression and *RHO* refers to the Spearman’s correlation. * denotes significance. In the second row, the corresponding FDR-corrected *p*-values are shown. A “<” preceding a *p*-value indicates that no permuted value exceeded the true value. Red boxes label the case where the quadratic regression *R*^2^ is significant, and the Spearman’s correlation is significant and positive. Blue boxes label the case where the quadratic regression *R*^2^ is significant, and the Spearman’s correlation is significant and negative. Green boxes label the case where the quadratic regression R^2^ is significant, but the Spearman’s correlation is not. Yellow boxes label the case where the quadratic regression *R*^2^ is not significant, but the Spearman’s correlation is.

We did not observe the predicted relationship for gF nor gC. For gC, we observed a significant positive trend for States 2–5, where higher controllability relates to higher positive regional importance only. For gF, while we observed significant quadratic relationships without significant trends for SC and States 2–3, the curves were concave—lower modal controllability regions were more predictive. State 6 exhibited a significant negative trend, where lower controllability relates to higher positive regional importance.

When examining this relationship for g, results were broadly consistent with some exceptions - SC showed significant quadratic relationships without significant monotonic trends (i.e., higher average controllability and lower modal controllability were associated with higher absolute regional importance), though strength showed no significant relationship. States 2–5 showed significant positive trends (i.e., regions with higher controllability and strength showed higher positive regional importance), though not for States 2 and 5 for modal controllability. State 6 showed significant negative trends (i.e., regions with lower controllability and strength showed higher positive regional importance), though not for average controllability.

### 3.7 Regional importance in predicting cognitive performance aligns with established brain maps

Regional importance in SC and dFC models predicting g visually highlighted a divergence between transmodal and unimodal networks. The principal gradient operationalizes this distinction, so we examined the relationship between regional importance and the principal gradient using quadratic regression and Spearman’s correlations.

Across dFC states only, average controllability and strength generally showed significant positive trends (i.e., higher positive regional importance in transmodal regions), and modal controllability generally showed significant negative trends (i.e., higher positive regional importance in unimodal regions) (Figure 11). This relationship with the principal gradient was robust to variation in thresholding, number of states, SC normalization, cognitive measure, and gender restriction (Supplementary Section 5). Interestingly, though, the relationship seems to be stronger for males.

**Figure 11:**
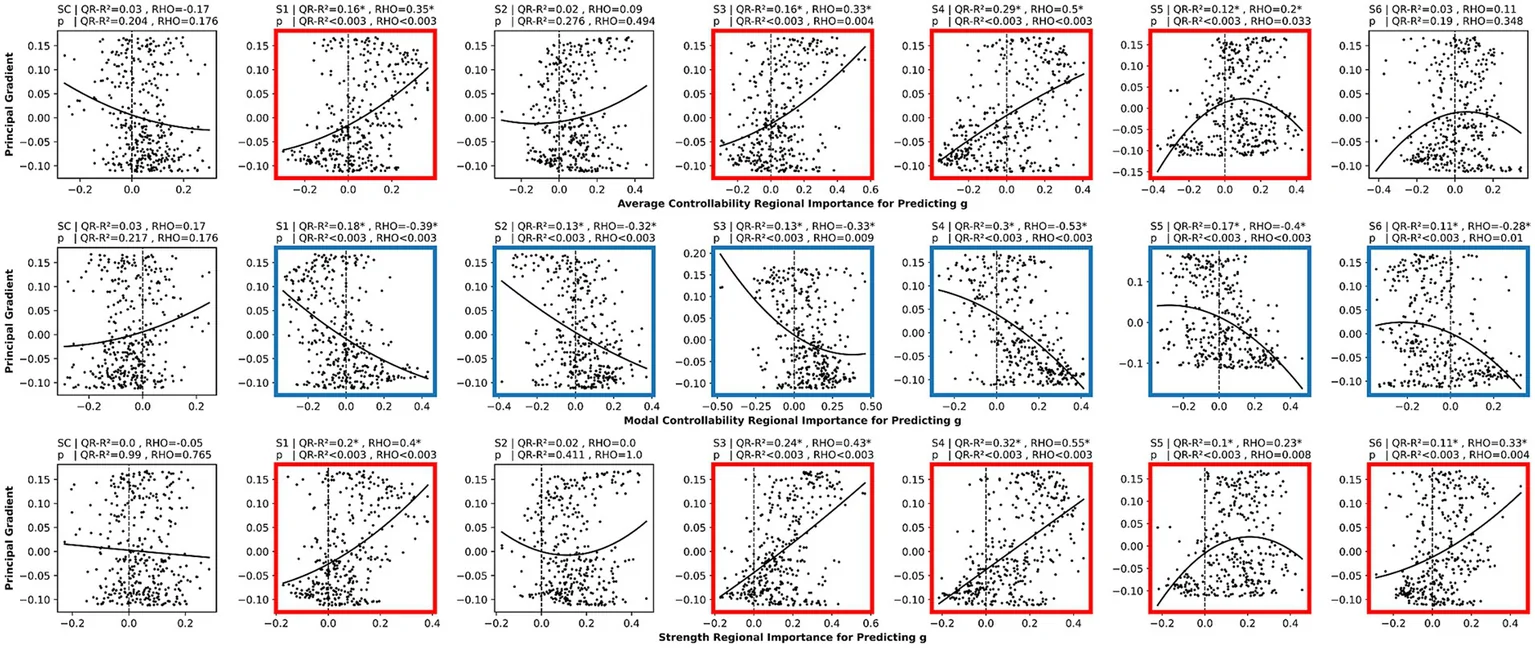
Scatterplots for the relationship between the regional importance for the prediction of *g* and principal gradient position. This characterizes the relationship between each controllability metric (average controllability, modal controllability, and strength) and regional importance in the corresponding model predicting *g*. Black dots correspond to regions, dotted black lines denote the positive and negative regional importance separation, and continuous black curves represent the quadratic regression model. In the first row of the titles, QR-*R2* refers to *R2* for the quadratic regression and *RHO* refers to the Spearman’s correlation. * Denotes significance. In the second row, the corresponding FDR-corrected *p*-values are shown. A “<” preceding a *p*-value indicates that no permuted value exceeded the true value. Red boxes label the case where the quadratic regression *R2* is significant, and the Spearman’s correlation is significant and positive. Blue boxes label the case where the quadratic regression *R2* is significant, and the Spearman’s correlation is significant and negative.

We further assessed the overlap between regions with significant regional importance in SC & dFC models predicting g and PFIT and MD regions using the Dice coefficient. Overlap with PFIT regions was significant for strength in State 4 (Table 3). Overlap with MD regions was significant for both controllability and strength for State 1, and for average controllability and strength for State 4 (Table 4).

**Table 3:** Dice Coefficient (DC) between regions with significant regional importance in the prediction of g and PFIT regions across connectivity matrices (columns).

	SC	S1	S2	S3	S4	S5	S6
Average							
	0.30 (*p* = 1.0)	0.38 (*p* = 0.53)	0.38 (*p* = 0.66)	0.38 (*p* = 1.0)	0.45 (*p* = 1.0)	0.41 (*p* = 1.0)	0.43 (*p* = 0.10)
Modal							
	0.30 (*p* = 1.0)	0.39 (*p* = 1.0)	0.38 (*p* = 0.96)	0.42 (*p* = 1.0)	0.34 (*p* = 1.0)	0.42 (*p* = 1.0)	0.47 (*p* = 0.75)
Strength							
	0.44 (*p* = 0.53)	0.35 (*p* = 0.079)	0.51 (*p* = 0.36)	0.50 (*p* = 0.32)	**0.57** **(*p* = 0.0052)**	0.52 (*p* = 0.50)	0.37 (*p* = 0.34)

Values with FDR-adjusted *p* < 0.05 appear in bold. Average and Modal refer to average and modal controllability, respectively.

**Table 4:** Dice coefficient (DC) between regions with significant regional importance in the prediction of g and MD regions across connectivity matrices (columns).

	SC	S1	S2	S3	S4	S5	S6
Average							
	0.20 (*p* = 0.66)	**0.46** **(*p* < 0.0030)**	0.084 (*p* = 1.0)	0.17 (*p* = 1.0)	**0.34** **(*p* < 0.0030)**	0.16 (*p* = 1.0)	0.21 (*p* = 0.65)
Modal							
	0.20 (*p* = 0.66)	**0.37** **(*p* < 0.0030)**	0.18 (*p* = 1.0)	0.17 (*p* = 1.0)	0.11 (*p* = 1.0)	0.17 (*p* = 1.0)	0.19 (*p* = 1.0)
Strength							
	0.24 (*p* = 0.54)	**0.53** **(*p* < 0.0030)**	0.17 (*p* = 1.0)	0.29 (*p* = 0.057)	**0.44** **(*p* < 0.0030)**	0.29 (*p* = 0.11)	0.24 (*p* = 0.25)

Values with FDR-adjusted *p* < 0.05 appear in bold. Average and Modal refer to average and modal controllability, respectively. A “<” preceding a *p*-value indicates that no permuted value exceeded the true value.

## 4 Discussion

This study investigated the prediction of gF and gC using modal and average controllability across multiple connectivity representations. It is worth emphasizing that the presumed link between cognitive effort and control energy in the Network Neuroscience Theory of Intelligence (Barbey, 2018) was not tested. Our findings inform the theory’s current operationalization, rather than the broader effort-based hypotheses regarding gF and gC. First, although most controllability studies focus on SC (Karrer et al., 2020; Gu et al., 2015; Wilcox et al., 2026; Anderson et al., 2023), we found that dFC substantially outperformed SC (and sFC) in predicting cognition. Second, we could not confirm the hypothesis that gC relates more strongly to average controllability and gF relates more strongly to modal controllability. Average controllability, modal controllability, and strength were highly correlated and showed similar predictive power. Instead, these network metrics best predicted g, rather than gF or gC. Notably, regional importance for predicting g using dFC aligned with the principal gradient, suggesting that control specialization along the cortical hierarchy supports general cognitive performance.

Consistent with evidence that sFC outperforms SC (Seguin et al., 2020; Parkes et al., 2021) and dFC outperforms sFC (Jia et al., 2014; Ramirez-Mahaluf et al., 2020), dFC outperformed SC in predicting cognitive performance here, whereas sFC did not offer a comparable advantage. This supports the idea that dFC may capture network characteristics important to the neurobiological basis of cognition (Deng et al., 2022; Papallo et al., 2025). Rather than treating controllability as time-invariant, state-based controllability might improve brain stimulation protocols aimed at effecting desirable brain activity (Muldoon et al., 2016). The use of controllability as a biomarker for development or disease could similarly benefit from a state-based approach (Tang et al., 2017; Parkes et al., 2021; He et al., 2022; Papallo et al., 2025). Nonetheless, structural and functional connectivity provide complementary information (Zimmermann et al., 2018; Dhamala et al., 2021; Wilcox et al., 2026), and dFC can be noise-sensitive (Hindriks et al., 2016; Laumann et al., 2017; Liégeois et al., 2017). Future research should look beyond coarse meso-to-macro MRI scales and parcellations (Rosen and Halgren, 2021), and explore alternative connectivity models, including effective connectivity (Deng et al., 2022), function-informed structural connectivity (Chu et al., 2018), polysynaptic routing (Seguin et al., 2022), and cortical geometry (Pang et al., 2023).

Although prior work has demonstrated meaningful differences between average controllability, modal controllability, and strength for specific scientific questions (Tang et al., 2017; Tu et al., 2018; Cornblath et al., 2019; Parkes et al., 2021; Deng et al., 2022; He et al., 2022), and our own study found differences in predictive performance and regional importance patterns, the differences in predictive performance for g, gF, and gC were not statistically significant. This held across modalities - we found no statistically significant differences for SC nor dFC alone. The close correspondence between average controllability, modal controllability, and strength calls into question the extent to which these metrics could be expected to provide unique information and differentiate behavioral constructs (Gu et al., 2015; Kenett et al., 2018; Rubinov, 2025), complicating any study design attempting to attribute them to gF and gC. Other studies have similarly reported near-inverse brain-behavior relationships between average and modal controllability (Kenett et al., 2018; Lee et al., 2020). This exaggerated correspondence may arise from the NCT model employed, as linear systems poorly capture distant connectivity dependencies that distinguish controllability (Tang et al., 2017; Deng et al., 2022). Of note, dFC exhibited higher correlations between network metrics than SC; this may inform future work deriving control metrics from FC. Alternatively, other measures of network control may be better suited to addressing these hypotheses. Controllability models an abstract scenario where sustained input to a single region drives the brain across its entire state space over unlimited time. Future work might instead implement optimal/minimum control frameworks, which may better capture how gC and gF relate to the ease of reaching specific brain states under more realistic constraints on control region selection and trajectory (Karrer et al., 2020).

Our findings indicate that controllability best predicts g, rather than gF and gC. Anderson et al. (2023) also observed significant associations between average controllability measured in SC and g in the anterior cingulate and inferior and middle temporal regions, with modal controllability showing similar associations. Wilcox et al. (2026) likewise reported that cognitive control, attention, language, and perceptual regions, also implicated in our findings, prominently featured in the modal controllability profile predicting g from SC. It is possible, however, that the weaker prediction of gF and gC observed may reflect the fidelity of our estimates. It may also reflect differences in measurement reliability - fewer tests were used to calculate all operationalizations of gF and gC in this study, which likely results in greater measurement error (Dubois et al., 2018). Better characterizing these cognitive domains [e.g., gF and gC derived from many tests or meta-analytical approaches; Agelink van Rentergem et al. (2020) may clarify this].

Controllability and regional importance for predicting g exhibited modality-specific relationships. In SC, regions with high average and low modal controllability showed the highest absolute regional importance, suggesting these regions are most influential. In dFC States 2–5, regions with higher controllability and strength generally showed greater predicted gains in cognitive performance as controllability and strength increased, suggesting that specialized connectivity (high values becoming higher, low values becoming lower) improves cognitive performance. We previously identified States 2–5 as cognitive-processing states where greater persistence predicts higher g (Ng et al., 2024). Here, we also found that States 2 and 5 contributed the most to the prediction of g. Specialized connectivity within these states might facilitate transitions into the cognitive-processing representations they support. In State 6, the opposite pattern emerged: within regions that had high controllability and strength relative to other regions, individuals with lower controllability and strength relative to their peers showed better cognitive performance. We previously characterized State 6 by the global anticoherence of the FPN, suggesting this state fails to support its usual task-general cognitive-control function–lower persistence predicted higher g. Connectivity facilitating transitions into this state’s representations may be maladaptive.

We found significant overlap between regions predictive of g and the regions implicated in MD and PFIT in States 1 and 4. For State 1, this overlap may arise because State 1, being the most frequent state, closely resembles sFC, and MD and PFIT were both derived from static brain measures (Crittenden et al., 2016; Jung and Haier, 2007). For State 4, this overlap may arise because the FPN, central to both MD and PFIT, shows particularly high strength in this state. This state dependence suggests that future work could explore incorporating dynamic connectivity into MD and PFIT.

Lastly, we investigated associations between regional importance and the principal gradient, extending prior work linking this gradient to controllability-strength separation (Parkes et al., 2021). Across dFC states, higher g was generally predicted by greater average controllability (and greater strength) in transmodal regions, alongside greater modal controllability (and lower strength) in unimodal regions. Transmodal regions engage in various tasks and include flexible hubs that adjust their connectivity to meet shifting cognitive demands (Anderson et al., 2013; Cole et al., 2013; Crossley et al., 2013; van den Heuvel et al., 2018). High average controllability in transmodal regions may facilitate the low-energy transitions between diverse brain states that such demands require (Lee et al., 2020). Conversely, unimodal sensorimotor regions may benefit from high modal controllability, which could enable high-energy transitions to novel brain states beyond their specialized functions, potentially supporting higher-order cognition.

### 4.1 Limitations and future directions

Several limitations stem from our methodological choices. First, results may differ under task states, which can act as cognitive “stress-tests” relative to resting-state (Sripada et al., 2020). Second, although fractional scaling has been validated (Donahue et al., 2016; Theodoni et al., 2021; Rosen and Halgren, 2021), its influence on control is not well characterized. Notably, however, unnormalized SC exhibited lower predictive power in our analyses. Better thresholding procedures may also clarify brain–behavior relationships. Third, we adopted a time-invariant, discrete-time NCT framework. Time-varying and continuous-time formulations may offer further insight (Karrer et al., 2020; Deng et al., 2022; Papallo et al., 2025). Fourth, interpretative limitations arise without experimental manipulation. As noted above, links between cognitive effort and control energy remain speculative. Regional inferences should also be approached with caution given the multifunctional nature of regions (Poldrack, 2011). Fifth, better feature selection may improve prediction. Reported results likely represent a lower bound. Sixth, we pooled sex in the main analysis. Minor differences emerged in gender-restricted analyses, hinting at underlying neurobiological differences that, if more deeply investigated, may help resolve this brain-behavior relationship. Future work could address these issues through task data and manipulation (e.g., cognitive effort), alternative connectivity and controllability formulations, brain stimulation, statistical model refinement, and examination of sex differences.

## 5 Conclusion

In summary, average and modal controllability significantly predicted gC and gF, with dFC-based controllability outperforming SC. However, both controllability metrics performed comparably to strength, and all three best predicted g. Specifically, higher g was predicted by higher average controllability in transmodal regions and higher modal controllability in unimodal regions during dFC states. These results suggest that future work on the neural bases of gC and gF should move beyond current controllability frameworks. Together, our findings advance empirical understanding of how network control relates to cognition and inform future neurobiological models of intelligence.

## Data Availability

Publicly available datasets were analyzed in this study. This data can be found here: https://www.humanconnectome.org/study/hcp-young-adult/document/1200-subjects-data-release.
