# Supplementary material for: Structural and dynamic functional controllability: shared variance between network control metrics limits attribution to distinct domains of intelligence
Source: Front Hum Neurosci. 2026 Oct 2;20:1920417. doi: 10.3389/fnhum.2026.1920417 (PMC13672590; doi:10.3389/fnhum.2026.1920417)
Supplement: Supplementary file 1 [file Data_Sheet_1.docx]

**Supplementary Figures**

**Figure S1. Percentage R^2^ variance contribution of each connectivity matrix for the prediction of g, gF, and gC with models using SC and dFC.** AC, MC, and S refer to average controllability, modal controllability, and strength respectively.

**Figure S2. Regional importance for the prediction of gF by average controllability, modal controllability, and strength in the SC & dFC model.** S# refers to one of 6 dFC states. Red labels positive importance, blue labels negative importance, and black labels non-significance.

**Figure S3. Regional importance for the prediction of gC by average controllability, modal controllability, and strength in the SC & dFC model.** S# refers to one of 6 dFC states. Red labels positive importance, blue labels negative importance, and black labels non-significance.

**Supplementary Tables**

**Table S1**

*Pearson’s correlation between group-averaged SC, sFC, and dFC connectivity matrices.*

|  | *SC* | *sFC* | *S1* | *S2* | *S3* | *S4* | *S5* | *S6* |
| --- | --- | --- | --- | --- | --- | --- | --- | --- |
| *SC* | -- | -- | -- | -- | -- | -- | -- | -- |
| *sFC* | 0.25 | -- | -- | -- | -- | -- | -- | -- |
| *S1* | 0.17 | 0.87 | -- | -- | -- | -- | -- | -- |
| *S2* | 0.16 | 0.85 | 0.62 | -- | -- | -- | -- | -- |
| *S3* | 0.23 | 0.66 | 0.46 | 0.44 | -- | -- | -- | -- |
| *S4* | 0.27 | 0.63 | 0.38 | 0.49 | 0.6 | -- | -- | -- |
| *S5* | 0.21 | 0.8 | 0.64 | 0.72 | 0.48 | 0.51 | -- | -- |
| *S6* | 0.21 | 0.75 | 0.59 | 0.57 | 0.54 | 0.47 | 0.39 | -- |

*Note.* S# refer to dFC states.

**Table S2**

*Difference in R^2^ for dFC and SC model comparisons for different cognitive measures (rows) and network metrics (columns).*

|  | *AC* | *MC* | *S* |
| --- | --- | --- | --- |
| *dFC vs SC* |  |  |  |
| *g* | **0.12**  **(p = 0.0021)** | **0.13**  **(p = 0.0018)** | **0.13**  **(p = 0.00086)** |
| *gF* | 0.053  (p = 0.087) | 0.053  (p = 0.087) | **0.064**  **(p = 0.0094)** |
| *gC* | **0.089**  **(p = 0.016)** | **0.092**  **(p = 0.0088)** | **0.11**  **(p = 0.0018)** |
| *SC & dFC vs dFC* |  |  |  |
| *g* | 0.012  (p = 0.37) | 0.010  (p = 0.44) | 0.0053  (p = 0.57) |
| *gF* | 0.023  (p = 0.16) | 0.021  (p = 0.16) | 0.015  (p = 0.20) |
| *gC* | 0.0035  (p = 0.80) | 0.0027  (p = 0.87) | -0.00069  (p = 0.93) |

*Note.* Values with FDR-adjusted p < 0.05 appear in bold. AC, MC, and S refer to average controllability, modal controllability, and strength respectively.

**Table S3**

*Difference in R^2^ for controllability and strength comparisons for different cognitive measures (columns) and modality combinations (rows).*

|  | *g* | *gF* | *gC* |
| --- | --- | --- | --- |
| *SC* |  |  |  |
| *Average* | 0.0071  (p = 0.74) | 0.00082  (p = 0.97) | 0.024  (p = 0.16) |
| *Modal* | 0.0057  (p = 0.79) | -0.000043  (p = 1.0) | 0.024  (p = 0.16) |
| *dFC* |  |  |  |
| *Average* | -0.00013  (p = 1.0) | -0.010  (p = 0.28) | 0.00098  (p = 0.97) |
| *Modal* | 0.0072  (p = 0.65) | -0.011  (p = 0.30) | 0.0040  (p = 0.79) |
| *SC & dFC* |  |  |  |
| *Average* | 0.0066  (p = 0.68) | -0.0025  (p = 0.92) | 0.0052  (p = 0.79) |
| *Modal* | 0.012  (p = 0.40) | -0.0046  (p = 0.82) | 0.0074  (p = 0.66) |

*Note.* Values with FDR-adjusted p < 0.05 appear in bold. Average and Modal refer to average and modal controllability, respectively.

**Table S4**

*Difference in R^2^ for cognitive variable comparisons (rows) in different modality combinations (columns).*

|  | *SC* | *dFC* | *SC & dFC* |
| --- | --- | --- | --- |
| *Average* |  |  |  |
| *g vs gF* | 0.0076  (p = 0.79) | **0.073**  **(p = 0.00013)** | **0.062**  **(p = 0.0021)** |
| *g vs gC* | 0.0076  (p = 0.80) | 0.037  (p = 0.20) | 0.045  (p = 0.13) |
| *Modal* |  |  |  |
| *g vs gF* | 0.0070  (p = 0.79) | **0.081**  **(p = 0.00043)** | **0.069**  **(p = 0.00086)** |
| *g vs gC* | 0.0056  (p = 0.87) | 0.041  (p = 0.15) | 0.048  (p = 0.077) |
| *Strength* |  |  |  |
| *g vs gF* | 0.0012  (p = 0.97) | **0.063**  **(p = 0.0021)** | **0.053**  **(p = 0.014)** |
| *g vs gC* | 0.024  (p = 0.20) | 0.038  (p = 0.20) | 0.044  (p = 0.10) |

*Note.* Values with FDR-adjusted p < 0.05 appear in bold. Average and Modal refer to average and modal controllability, respectively.

**Supplementary Sections**

**Supplementary Section 1. Cognitive metrics**

The estimation of g is comparatively straightforward. We conducted a bifactor CFA with the same 10 cognitive tests outlined by [Dubois et al. (2018)](https://paperpile.com/c/8cWNvA/xpOI). In a CFA, the factor structure is specified and cognitive test scores are not allowed to cross-load on different factors. This is in contrast to an EFA, where the number of factors are specified and the factor structure is estimated from the underlying latent variance. It is usually preferable to derive scores from a CFA, but there is not much difference between CFA and EFA for estimating g. Similar to their procedure, to improve the estimation of the cognitive factor structure, we used all 1192 participants with all 10 cognitive test scores. This sample is slightly different, as they had thresholded by additional criteria including other behavioral scores.

The estimation of gF and gC is more contentious. In a bifactor analysis, g is estimated and the remaining variance is assigned to other factors. One important consideration is that these factors are constrained to be orthogonal to both g and other factors. It is difficult to interpret these factors originating from the bifactor CFA for g as gF or gC, as gF and gC as constructs seem to generally load onto g in prior cognitive factor analyses. A higher-order factor analysis could be conducted, where a first-order factor analysis is conducted on the cognitive tests, and a second-order factor analysis is conducted on the first factors. However, contemporary factor analyses suggest that many more second-order (i.e., below g) factors than just gF and gC [(Agelink van Rentergem et al. 2020)](https://paperpile.com/c/8cWNvA/LMzM). In line with this, in first-order EFA of the same data, PMAT24 and LineOrient load highly on the same factor across different choices for the number of factors. Both tests index visuospatial ability, so the corresponding factor may index a second-order factor for visuospatial ability rather than gF. Furthermore, CardSort, Flanker, and ProcSpeed also load highly on the same factor across choices of number. Each of these tests take into account reaction speed in the scoring, so the corresponding factor may index a second-order factor for reaction speed rather than gF. The number of cognitive tests in HCP data may not be enough to capture the factor structure required to estimate gF and gC by EFA. Another choice may be CFA, but there may not be enough background literature to support our choice of factor structure. In fact, in many prior studies investigating gF and gC in HCP data, factor scores are not used. One choice may be to use the NIH toolbox measures, which estimates gC by averaging PicVoc and ReadVoc, and gF by averaging CardSort, Flanker, PicSeq, ListSort, and ProcSpeed. Another choice is to estimate gC and gF with PicVoc and PMAT24 respectively [(Hearne et al. 2016; Greene et al. 2018; Tadayon et al. 2020)](https://paperpile.com/c/8cWNvA/8sEdp+Dj4oZ+4oagL). PicVoc should not differ substantially from NIH gC, as both ReadVoc and PicVoc measure vocabulary ability. However, given that NIH gF averages many cognitive tests that measure different cognitive abilities and may load better on other cognitive factors, it may be considered a less direct measure of gF [(Tadayon et al. 2020)](https://paperpile.com/c/8cWNvA/4oagL).

Taking this into consideration, in the main analysis, we measure g from CFA using the bifactor structure specified by [Dubois et al. (2018)](https://paperpile.com/c/8cWNvA/xpOI) and estimate fit metrics to ensure that the fit is still adequate with the new sample, gF with PMAT24, and gC with PicVoc. Post-hoc, we assessed g derived from EFA of the same 10 tests, g derived from PCA of the same 10 tests, NIH gF, and NIH gC to investigate whether average and modal controllability better predict these estimates of g, gF, and gC. These results are found in Supplementary Section 5.

**Supplementary Section 2. sFC comparisons**

Given that dFC predicted cognitive performance better than SC across multiple cognitive measures, is the difference due to a difference between functional and structural modalities, or the dynamics? If it were a difference between modalities, we might also expect to see a higher contribution to the prediction by sFC than SC for g, gF, and gC like dFC, the difference between dFC and SC to also be observed between sFC and SC, no difference between the predictiveness of dFC and sFC, and no difference between the predictiveness of combined SC & dFC and SC & sFC models. We found that sFC did not contribute as high a percentage as dFC when placed in the same model as sFC (with SC even contributing more to the prediction of gF) (Figure 8). We also did not find that sFC predicted cognition significantly better than SC unlike dFC (Supplementary Section 2 - Table 1). On the other hand, dFC predicted significantly better than sFC, and SC & dFC predicted significantly better than SC & sFC for g, gF, and gC. This indicates that structural-functional differences were not fully driving the improvement in prediction by dFC over SC, suggesting that the time-varying nature of dFC is important.

**Supplementary Section 2 - Table 1**

*Difference in R^2^ for sFC model comparisons for different cognitive measures (rows) and network metrics (columns).*

|  | *AC* | *MC* | *S* |
| --- | --- | --- | --- |
| *sFC vs SC* |  |  |  |
| *g* | 0.014  (p = 0.72) | 0.0074  (p = 0.87) | 0.040  (p = 0.16) |
| *gF* | -0.014  (p = 0.68) | -0.019  (p = 0.57) | -0.0038  (p = 0.92) |
| *gC* | 0.0023  (p = 0.97) | 0.0015  (p = 0.97) | 0.047  (p = 0.070) |
| *dFC vs sFC* |  |  |  |
| *g* | **0.10**  **(p = 0.00018)** | **0.12**  **(p = 0.000014)** | **0.086**  **(p = 0.0011)** |
| *gF* | **0.068**  **(p = 0.0021)** | **0.073**  **(p = 0.00086)** | **0.068**  **(p = 0.0018)** |
| *gC* | **0.087**  **(p = 0.0018)** | **0.090**  **(p = 0.00044)** | **0.065**  **(p = 0.0021)** |
| *SC & dFC vs SC & sFC* |  |  |  |
| *g* | **0.094**  **(p = 0.00071)** | **0.10**  **(p = 0.00018)** | **0.081**  **(p = 0.0014)** |
| *gF* | **0.058**  **(p = 0.0034)** | **0.063**  **(p = 0.0021)** | **0.060**  **(p = 0.0021)** |
| *gC* | **0.069**  **(p = 0.0090)** | **0.074**  **(p = 0.0021)** | **0.066**  **(p = 0.0021)** |

*Note.* Values with FDR-adjusted p < 0.05 appear in bold. AC, MC, and S refer to average controllability, modal controllability, and strength respectively.

**Supplementary Section 3. Thresholding**

A common approach is to threshold by the strength of the group-averaged connection weight [(Roberts et al. 2017)](https://paperpile.com/c/8cWNvA/tM62b). This can remove true weak long-range connections. Given that we aim to investigate a controllability metric specifically sensitive to weak long-range ties, we used consistency-based thresholding. The consistency of connection weights across participants is calculated (i.e., the coefficient of variation of the connection weight across participants) and a proportion of connections with the most consistent weights across participants is kept. If a weight is weak but consistent across participants, this supports the existence of the connection.

Deterministic tractography generally produces connectomes with low network densities, such as 0.18-0.23 (i.e. 18-23% of all possible connections exist) [(Mori et al. 2008; Tang et al. 2017; Cui et al. 2019)](https://paperpile.com/c/8cWNvA/ohx6b+8O5r+CMX6G). However, tracer studies point towards 0.66-0.73 dense connectomes in mammals [(Markov et al. 2014; Ypma & Bullmore 2016)](https://paperpile.com/c/8cWNvA/dveGG+6VhiJ). Thus, we chose a balanced 0.5 connectome density in our main analysis.

Completely disconnected regions of the brain are biologically implausible in a healthy sample and this exclusion might have influence on distant regions, but our thresholding procedure in the main manuscript does not prevent this. To examine this issue, we also adapted a minimum spanning tree (MST) procedure to produce the minimum connections with the highest consistency required to fully connect the network [(Luppi & Stamatakis 2021)](https://paperpile.com/c/8cWNvA/T9F49), then iteratively added edges until the network reached the desired network density.

Results of different thresholding are found in Supplementary Section 5.

**Supplementary Section 4. Network Control Theory**

NCT can be used to describe how activity theoretically evolves from an empirically derived network. Linear models approximate true nonlinear brain dynamics to identify trends [(Gu et al. 2015; Karrer et al. 2020)](https://paperpile.com/c/8cWNvA/Qbh9O+ZNhOD). We used the linear time-invariant discrete time network control model x(t + 1) = Ax(t) + Bu(t). N is the number of brain regions, M is the number of control nodes, A is the N x N adjacency matrix (i.e. SC, sFC, or dFC), x(t) is the N x 1 vector of brain activity which represents the state at time t, B is the N x M control input matrix which consists of columns of the identity matrix with 1 for the control nodes and 0 otherwise, and u(t) is the M x 1 vector of input energy at each control node. In other words, A can be considered a transformation, such that x(t) is transformed into x(t + 1) by matrix multiplication with A with changes generated by adding Bu(t).

The diagonals of each A were set to zero because self-loops were not of interest. To avoid infinite growth of activity which is neurobiologically impossible, we normalized the system such that activity decays to zero over time by producing A_norm_ = A / λ(A)_max_ + 1, where λ(A)_max_ is the largest positive eigenvalue of A.

**Global controllability**

Average and modal controllability measure how well input energy into the region drives towards low energy required and high energy required states respectively. A prerequisite for such calculations is that the system is globally controllable, such that input into the region can drive any arbitrary state into any other state [(Wilmskoetter et al. 2022)](https://paperpile.com/c/8cWNvA/ylD6Q).

Global controllability can be probed using the controllability Gramian, a matrix constructed from elements of the network control model [(Gu et al. 2015)](https://paperpile.com/c/8cWNvA/Qbh9O). Smaller eigenvalues of this matrix means that more energy is required for some activity states, such that the smallest eigenvalue is inversely proportional to the largest energy required to reach an activity state. Consequently, the smallest eigenvalue must be greater than zero for global controllability. Calculation of the controllability Gramian will also produce errors if the system is not globally controllable. We calculated the controllability Gramian for every network control model generated, and discarded regions which failed the test from further prediction models. We found that this was reasonable, as the regions which failed the test were ones that were disconnected from the rest of the network and generated invariant average and modal controllability error values across subjects.

**Average controllability and modal controllability**

Average controllability measures the capacity for a region to drive brain activity towards low energy required states. It is estimated as the trace (the sum of the eigenvalues) of the controllability Gramian. As mentioned, smaller eigenvalues indicate greater energy required for some states. Thus, the trace quantifies the average input energy required for all possible activity states. This encodes the system impulse response. The higher this value, the more activity states can be reached with low input energy.

Modal controllability measures the capacity for a region to drive brain activity towards high energy required states. As mentioned, A can be considered a transformation. The eigenvectors of A describe the orthogonal modes of transformation. Another global controllability test is the PBH test. A region’s eigenvector entries determine whether the mode described by the eigenvector is controllable by that region - zero means that it is not controllable. By extension, larger values mean better control. Eigenvalues describe the rate of decline along that mode - smaller eigenvalues indicate fast decaying modes which are more difficult to control because input energy has a shorter term impact. Modal controllability is estimated as a sum of the region’s eigenvector entries (control capacity) weighted towards smaller eigenvalues (difficult to control modes). The higher this value, the better the high input energy activity states are controlled.

**Supplementary Section 5. Parameter comparisons**

We compared R^2^ for predicting g using SC, dFC, and SC & dFC models across different thresholds, k, SC normalization, and procedures used to calculate g to assess whether our finding, that dFC outperforms SC, was robust across parameters (Supplementary Section 5 - Figure 1). Across parameters, the dFC model generally outperformed the SC model, whereas the SC & dFC model showed performance comparable to dFC.

We also compared R^2^ for predicting g using average controllability, modal controllability, and strength across different thresholds, k, SC normalization, and procedures used to calculate g to evaluate whether our finding, that controllability and strength exhibits similar performance, was robust across parameters (Supplementary Section 5 - Figure 2). Average controllability, modal controllability, and strength exhibited similar predictive performance across parameters.

Furthermore, we compared R^2^ for predicting g, gF, and gC across different thresholds, k, SC normalization, and procedures used to calculate g, gF, and gC to determine whether our finding, that g is better predicted than gF and gC, was robust across parameters (Supplementary Section 5 - Figure 2). Across parameters, g was better predicted than both gF and gC.

We also assessed R^2^ for predicting g using average controllability, modal controllability, and strength and g, gF, and gC across different thresholds for SC specifically (Supplementary Section 5 - Figure 3), in case our results were specific to controllability calculated from dFC. We found that the predictive power of average and modal controllability was relatively stable across thresholds, while strength varied more. Strength had the strongest predictive power around the 50-60% group consistency threshold, supporting its use in the main manuscript, and lower predictive power at more extreme thresholds. This suggests findings in prior literature comparing controllability and strength may be affected by the network density of the connectivity matrix used. g was also better predicted than gF and gC in SC.

We also compared the Spearman’s correlation between the principal gradient and feature importance from the SC & dFC model within each state across different thresholds, k, SC normalization, and cognitive measures used to calculate g to assess whether our main finding, positive correlations for average controllability and negative correlations for modal controllability in dFC states, was robust across parameters (Supplementary Section 5 - Figure 4). Across most parameters, average controllability showed positive correlations and modal controllability showed negative correlations, consistent with the main analysis. Some exceptions were observed at more extreme thresholds and k, particularly for average controllability in States 2 and 6, which also did not show statistically significant relationships with the principal gradient in the main analysis.

Across models, the group consistency threshold of 50% used in the main analysis generally yielded reasonable predictive performance relative to other choices, supporting its use in the primary analyses. Interestingly, R^2^ values tended to increase with k, suggesting that finer-grained temporal characterizations of brain network dynamics may improve the predictive utility of controllability measures for general cognitive domains. Notably, the main analysis choice of k = 6 also yielded reasonable predictive performance relative to other choices. We further found that g derived using EFA and PCA generally yielded predictive accuracies similar to those obtained using CFA in the main analysis. Likewise, NIH-defined gF and gC did not substantially improve predictive performance overall. NIH-defined gF was predicted poorly compared to gF derived from PMAT24 - this may be because it represents a less direct measure of fluid intelligence [(Tadayon et al. 2020)](https://paperpile.com/c/8cWNvA/4oagL).

We also repeated the analysis using only data from participants of each gender (Supplementary Section 5 - Figure 5). While some differences emerged (e.g., controllability calculated from SC generally performed worse at predicting cognitive performance in males compared to females, the divergence between the correlation between the principal gradient and average and modal controllability was generally larger in males compared to females), our main findings were generally robust to gender: dFC outperformed SC in predicting g, and the SC & dFC model performed comparably to dFC alone. Controllability and strength also showed similar predictive performance. When comparing the SC & dFC model's performance across cognitive variables, g was again better predicted than both gF and gC. Finally, correlations between the principal gradient and feature importance from the SC & dFC model remained generally positive for average controllability and negative for modal controllability.

Lastly, results for the MST procedure were highly consistent for our main model (i.e., prediction of g using SC & dFC with 50% network density). R^2^ and variance decompositions were nearly identical (Supplementary Section 5 - Table 1). Moreover, Pearson’s correlations between regional importance scores to prediction exceeded 0.9999 across all matrices. This consistency should be expected, as only 10 regions for State 1 were removed for the main model which correspond to areas prone to signal dropout near the orbitofrontal cortex, where connections involving these regions likely carry spurious values (Supplementary Section 5 - Figure 6).

**Supplementary Section 5 - Figure 1. SC and dFC R^2^ comparison across different parameters for the prediction of g by average controllability (AC), modal controllability (MC), and strength (S). (**A) Comparison between percentage thresholding by consistency and strength. (B) Comparison between different values of k. (C) Comparison between fractional scaling (FPT) and raw (RAW) streamline counts for SC. (D) Comparison between g calculated from CFA, EFA, and PCA.

**Supplementary Section 5 - Figure 2. g, gF, and gC R^2^ comparison across different parameters for average controllability (AC), modal controllability (MC), and strength (S) in the SC & dFC model. (**A) Comparison between percentage thresholding by consistency and strength. (B) Comparison between different values of k. (C) Comparison between fractional scaling (FPT) and raw (RAW) streamline counts for SC. (D) Comparison between g calculated from CFA, EFA, and PCA, gF calculated as PMAT24 and NIH-defined gF, and gC calculated as PicVoc and NIH-defined gC.

**Supplementary Section 5 - Figure 3. R^2^ comparison across different thresholds for the SC model. (**A) Comparison for average controllability (AC), modal controllability (MC), and strength (S). (B) Comparison for g, gF, and gC.

**Supplementary Section 5 - Figure 4. Spearman’s correlation between the principal gradient and feature importance in each state across different parameters for the prediction of g with average controllability (AC), modal controllability (MC), and strength (S) in the SC & dFC model. (**A) Comparison between percentage thresholding by consistency and strength. (B) Comparison between different values of k. (C) Comparison between fractional scaling (FPT) and raw (RAW) streamline counts for SC. (D) Comparison between g calculated from CFA, EFA, and PCA.

**Supplementary Section 5 - Figure 5. Comparison between genders. (**A) R^2^ for the prediction of g with average controllability (AC), modal controllability (MC), and strength (S) in the SC & dFC, dFC, and SC models. (B) R^2^ for the prediction of g, gF, and gC with average controllability, modal controllability, and strength in the SC & dFC model. (C) Spearman’s correlation between the principal gradient and feature importance in each state for the prediction of g with average controllability, modal controllability, and strength in the SC & dFC model.

**Supplementary Section 5 - Figure 6. Disconnected regions at 50% group consistency thresholding in State 1.** Pink regions correspond to regions which were disconnected and thus removed from the analysis.

**Supplementary Section 5 - Table 1**

*Model R^2^ and percentage contributions for different cognitive measures (rows) and network metrics (columns) thresholded with and without MST for the SC & dFC model.*

|  | *Average* | *Modal* | *Strength* |
| --- | --- | --- | --- |
| *Without MST* |  |  |  |
| *g* | 0.16  SC = 14%  dFC = 86% | 0.17  SC = 13%  dFC = 87% | 0.15  SC = 8%  dFC = 92% |
| *gF* | 0.099  SC = 28%  dFC = 72% | 0.097  SC = 27%  dFC = 73% | 0.10  SC = 20%  dFC = 80% |
| *gC* | 0.12  SC = 10%  dFC = 90% | 0.12  SC = 10%  dFC = 90% | 0.11  SC = 2%  dFC = 98% |
| *With MST* |  |  |  |
| *g* | 0.16  SC = 14%  dFC = 86% | 0.17  SC = 13%  dFC = 87% | 0.15  SC = 8%  dFC = 92% |
| *gF* | 0.097  SC = 28%  dFC = 72% | 0.096  SC = 27%  dFC = 73% | 0.10  SC = 21%  dFC = 79% |
| *gC* | 0.11  SC = 10%  dFC = 90% | 0.12  SC = 10%  dFC = 90% | 0.11  SC = 2%  dFC = 98% |

*Note.* Average and Modal refer to average and modal controllability, respectively.

**Supplementary References**

Cui, L.-B., Wei, Y., Xi, Y. B., Griffa, A., de Lange, S. C., Kahn, R. S., et al. (2019). Connectome-based patterns of first-episode medication-naïve patients with schizophrenia. S*chizophr. Bull.* 45, 1291–1299. doi: 10.1093/schbul/sbz014

Luppi, A. I., and Stamatakis, E. A. (2021). Combining network topology and information theory to construct representative brain networks. *Netw. Neurosci.* 5, 96–124. doi: 10.1162/netn_a_00170

Markov, N. T., Ercsey-Ravasz, M. M., Ribeiro Gomes, A. R., Lamy, C., Magrou, L., Vezoli, J., et al. (2014). A weighted and directed interareal connectivity matrix for macaque cerebral cortex. *Cereb. Cortex* 24, 17–36. doi: 10.1093/cercor/bhs270

Mori, S., Oishi, K., Jiang, H., Jiang, L., Li, X., Akhter, K., et al. (2008). Stereotaxic white matter atlas based on diffusion tensor imaging in an ICBM template. *NeuroImage* 40, 570–582. doi: 10.1016/j.neuroimage.2007.12.035

Ypma, R. J. F., and Bullmore, E. T. (2016). Statistical analysis of tract-tracing experiments demonstrates a dense, complex cortical network in the mouse. *PLoS Comput. Biol.* 12:e1005104. doi: 10.1371/journal.pcbi.1005104
